# An Overview of the Synthesis of 3,4-Fused Pyrrolocoumarins of Biological Interest

**DOI:** 10.3390/molecules29122748

**Published:** 2024-06-09

**Authors:** Eleni Kapidou, Konstantinos E. Litinas

**Affiliations:** Laboratory of Organic Chemistry, Department of Chemistry, Aristotle University of Thessaloniki, 54124 Thessaloniki, Greece; ekapidou@chem.auth.gr

**Keywords:** 3,4-fused pyrrolocoumarin, aminocoumarin, MCR reaction, metal-catalyzed reactions

## Abstract

3,4-Fused pyrrolocoumarins, synthetically prepared or naturally occurring, possess interesting biological properties. In this review, the synthetic strategies for the synthesis of the title compounds are presented along with their biological activities. Two routes are followed for that synthesis. In one, the pyrrole ring is formed from coumarin derivatives, such as aminocoumarins or other coumarins. In the other approach, the pyranone moiety is built from an existing pyrrole derivative or through the simultaneous formation of coumarin and pyrrole frameworks. The above syntheses are achieved via 1,3-dipolar cycloaddition reactions, Michael reaction, aza-Claisen rearrangement reactions, multi-component reactions (MCR), as well as metal-catalyzed reactions. Pyrrolocoumarins present cytotoxic, antifungal, antibacterial, α-glucosidase inhibition, antioxidant, lipoxygenase (LOX) inhibition, and fluorescent activities, as well as benzodiazepine receptor ability.

## 1. Introduction

**Figure 1:**
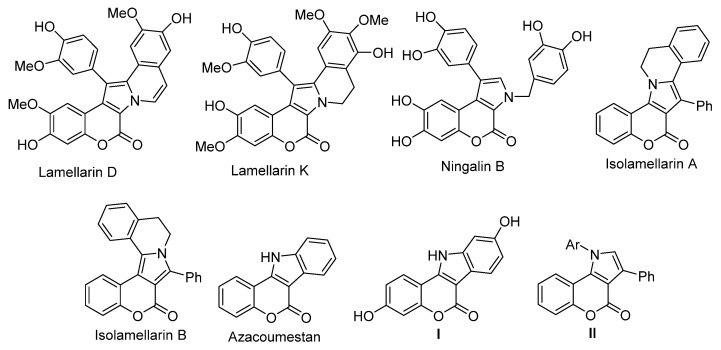
Fused pyrrolocoumarins found in nature and/or presenting diverse biological activities.

Coumarin derivatives are widely found in nature, coming as secondary metabolites from trees, plants, and microorganisms [1,2,3,4,5,6,7,8,9]. Both natural and synthetic coumarin derivatives present a wide range of biological properties [10,11,12,13,14,15], such as anticancer [16,17], anti-HIV [18,19], anti-SARS-CoV-2 [3], antidiabetic [20,21], anti-inflammatory [22,23], anticoagulant [24,25], antibiotic [26,27], antitubercular [28,29], neuroprotective [30,31], anti-infectious hematopoietic necrosis virus activities (IHNV) [32], etc. Fused coumarins possess also biological activities, and many of them, like furocoumarins [33,34], pyranocoumarins [35] or pyridocoumarins [36] are isolated from nature. Fused pyrrolocoumarins display interesting biological properties, such as cytotoxic [37,38,39,40,41,42], antibacterial [42], anti-HIV [37,42,43,44,45], multidrug resistance reversal (MDR) [37,42,45], antibiotic [42], antioxidant [42,46,47], antitumor [45], and anti-inflammatory activities [42,46,47]. Some of them act also as benzodiazepine receptor (BZR) ligands [48,49], angiogenesis inhibitors [50], DYRK1A inhibitors [45,51], Wnt signaling inhibitors [52] or fluorescent probes for live cell imaging [53]. Some of the fused pyrrolo[2,3-c]coumarins, the classes of lamellarins or ningalins, were isolated from sea snails and squirts. Especially, lamellarin D (Figure 1) was isolated from methanol extracts of marine prosobranch mollusks *Lamellaria* sp. by Pallaou [54]. Lamellarin K was isolated from the dichloromethane-methanol extracts of an ascidian *Didemnum obscurum* collected from Tamilnadu, India [55]. Ningalin B was obtained from methanol/chloroform extracts of the ascidian of the genus *Didemnum* collected in western Australia [56]. The fused pyrrolo[3,2-c]coumarin and pyrrolo[3,4-c]coumarin are the core moieties of biologically active isolamellarin A, azacoumestans and isolamellarin B, respectively (Figure 1) [57,58,59]. 3,9-Dihydroxyazacoumestan (I) is an angiogenesis inhibitor [50], while 1-aryl-3-phenylpyrrolo[3,2-c]coumarins II are benzodiazepine receptor ligands [48,49]. In the literature, many reviews have been published concerning the synthesis and biological activities of coumarin and fused coumarin derivatives, while fused pyrrolocoumarins have appeared in some reviews [1,14,45,60,61,62,63,64,65,66,67] and in a few specific reviews [57,58,68]. This review will present an overview of the synthesis and biological evaluation of fused pyrrolo[*c*]coumarins.

## 2. Synthetic Strategies for the Preparation of [3,4]-Fused Pyrrolocoumarin Derivatives

The synthesis of [3,4]-fused pyrrolocoumarins has been achieved through two main routes. The formation of pyrrole moiety starting from a coumarin derivative is the first, while the second is the formation of pyranone ring starting from a pyrrole derivative.

### 2.1. Pyrrole Ring Formation

As starting materials for the formation of pyrrole moiety, different fused heterocyclic coumarin derivatives have been used: 3- or 4-aminocoumarins or hydroxycoumarins, chlorocoumarins, or other coumarin derivatives [69]. Multicomponent reactions (MCR) or metal-catalyzed reactions have been utilized for the synthesis of pyrrole ring of [3,4]-fused pyrrolocoumarins. 

#### 2.1.1. Synthesis from Aminocoumarin Derivatives

**Scheme 1:**
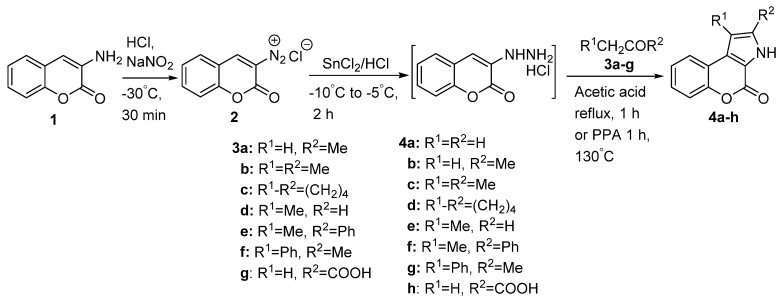
Synthesis of benzopyrano[3,4-b]pyrrol-4-ones **4a**–**h** via Fischer indolization reaction.

In 1978, Khan and de Brito Morley reported for the first time the synthesis of substituted fused pyrrolo[3,4-b]coumarins **4b**–**h** via the Fischer indolization of in situ prepared coumarin-3-hydrazine hydrochloride with carbonyl compounds **3a**–**g** in 33–51% yield [70]. The intermediate hydrazine hydrochloride was prepared by the reduction, with stannous chloride, of 3-coumarin diazonium salt (**2**) synthesized by diazotization of 3-aminocoumarin (**1**) at −30 °C (Scheme 1).

**Scheme 2:**
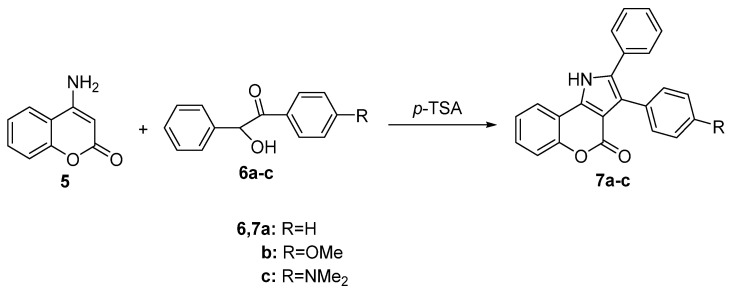
Synthesis of 2,3-diaryl-4(*H*)-oxo[1]benzopyrano[4,3-*b*]pyrroles **7a**–**c** from 4-aminocoumarin and benzoin.

Joshi et al. presented the synthesis of the 2,3-diaryl-4(*H*)-oxo[1]benzopyrano[4,3-*b*]pyrroles **7a**–**c** by the reaction of 4-aminocoumarin (**5**) with benzoins **6a**–**c** in the presence of *p*-toluene sulfonic acid (Scheme 2). These novel compounds produced a strongly fluorescent green solution in alkali [71].

**Scheme 3:**
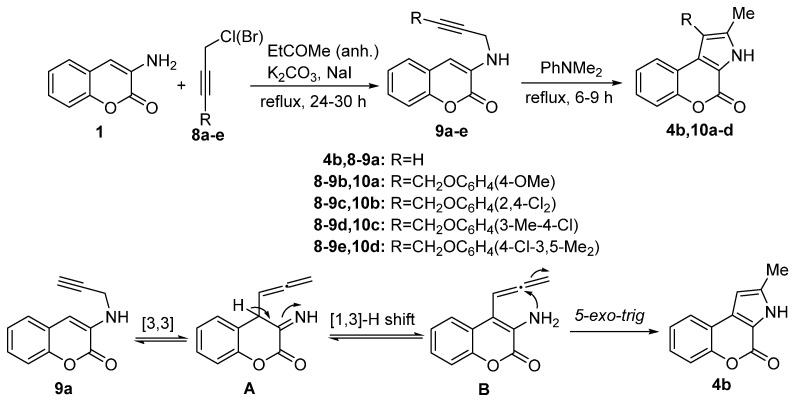
Synthesis of benzopyrano[3,4-*b*]pyrrol-4-ones **4b**, **10a**–**d** via aza-Claisen rearrangement of propargylamino compounds **9a**–**e**.

Majumdar and Chattopadhyay prepared the pyrrolocoumarin derivatives **4b**, **10a**–**d** in excellent yield of 90–94% from the regioselective aza-Claisen rearrangement of 3-*N*-propargylaminocoumarin (**9a**) and 3-*N*-(aryloxybut-2-ynyl)aminocoumarins **9b**–**e** (Scheme 3). An initial [3,3]-sigmatropic rearrangement of propargylamine moiety of **9a** gave allene intermediate **A**, followed by a tautomerism to amine **B**. A subsequent 5-*exo-trig* cyclization resulted in the final product **4b**. Starting compounds **9a**–**e** was synthesized by refluxing 3-aminocoumarin (**1**) with propargyl bromide (**8a**) or 1-aryloxy-4-chlorobut-2-ynes **8b**–**e**, respectively, in anhydrous ethyl methyl ketone [72].

**Scheme 4:**
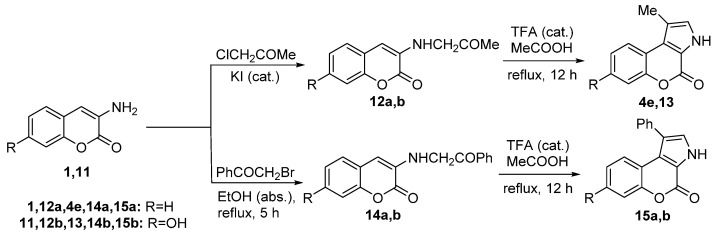
Synthesis of chromeno[3,4-*b*]pyrrol-4(3*H*)-ones **4e**, **13** and **15a**,**b** by the cyclization of 3-*N*-ketonylcoumarins in the presence of TFA.

Soman et al. synthesized chromeno[3,4-*b*]pyrrol-4(3*H*)-ones **4e**, **13** and **15a**,**b** in 28–68% yield through cyclization, in the presence of catalytic amount of TFA, of 3-*N*-ketonylcoumarins **12a**,**b** and **14a**,**b**, respectively, which were obtained by the reaction of 3-aminocoumarins **1**, **11** with a-haloketones (Scheme 4). The compounds **4e**, **13** was tested for their cytotoxic activity using the MTT [3-(4,5-dimethylthiazo-2-yl)-2,5-diphenyl-2*H*-tetrazolium bromide] against cell lines of colon, lung, and breast cancer. Both compounds showed good activity against all the above cell lines [39].

**Scheme 5:**
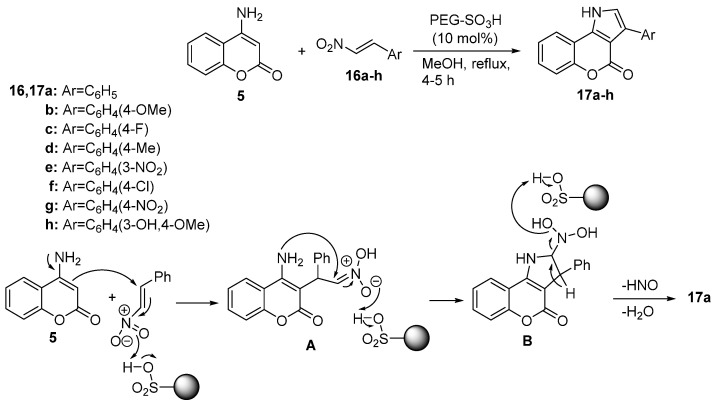
Synthesis of 3-aryl-4(*H*)-oxo[1]benzopyrano[4,3-*b*]pyrroles **17a**–**h** from 4-aminocoumarin and β-nitrostyrenes under PEG-SO_3_H catalysis.

Paul and Das reported the synthesis of 3-aryl-4(*H*)-oxo[1]benzopyrano[4,3-*b*]pyrroles **17a**–**h** from 4-aminocoumarin (**5**) and β-nitrostyrenes **16a**–**h** in the presence of the polymer-supported catalyst PEG-SO_3_H in 72–85% yield [73]. In the proposed mechanism, PEG-SO_3_H catalyzed the Michael addition of **5** to the β-nitrostyrene. Subsequent intramolecular amine attack of the Michael adduct **A** afforded the cyclized intermediate **B**. Elimination of water and nitroxyl molecules resulted in the final product (Scheme 5). 

**Scheme 6:**
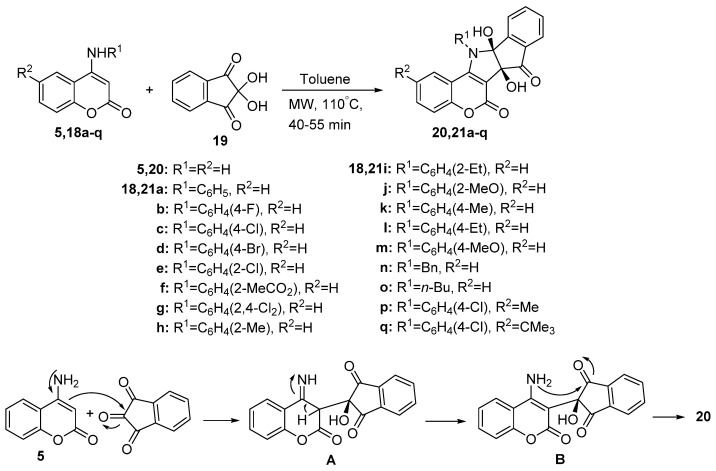
Synthesis of dihydrochromenoindeno[1,2-*b*]pyrroles **20**, **21a**–**q** from 4-aminocoumarins and ninhydrin.

Chen et al. presented a regioselective synthesis of dihydrochromenoindeno[1,2-*b*]pyrroles **20**, **21a**–**q** in 70–92% yield via the reaction of N-substituted 4-aminocoumarin derivatives **5**, **18a**–**q** with ninhydrin (**19**) under microwave irradiation without the presence of a catalyst [74]. Nucleophilic addition of 4-aminocoumarin (**5**) to the more electrophilic middle carbonyl of indanetrione, which is in equilibrium with ninhydrin, gave the intermediate **A**. Tautomerization of **A** resulted in **B**. The addition of amine to one of the carbonyls led to the final product **20** (Scheme 6). 

**Scheme 7:**
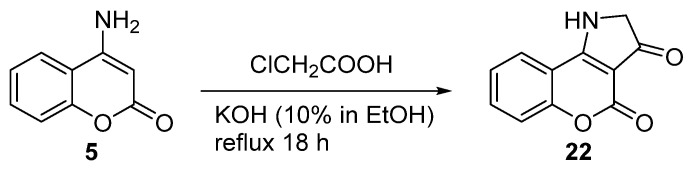
Synthesis of 1,2-dihydrochromeno[4,3-*b*]pyrrole-3,4-dione (**22**).

Ibrahim et al. reported that the reaction of 4-aminocoumarin (**5**) with chloroacetic acid resulted in 1,2-dihydrochromeno[4,3-*b*]pyrrole-3,4-dione (**22**) in 58% yield (Scheme 7). This compound was studied for cytotoxic activity and found to be 1.5-fold more toxic than the standard 5-fluorouracil [75].

**Scheme 8:**
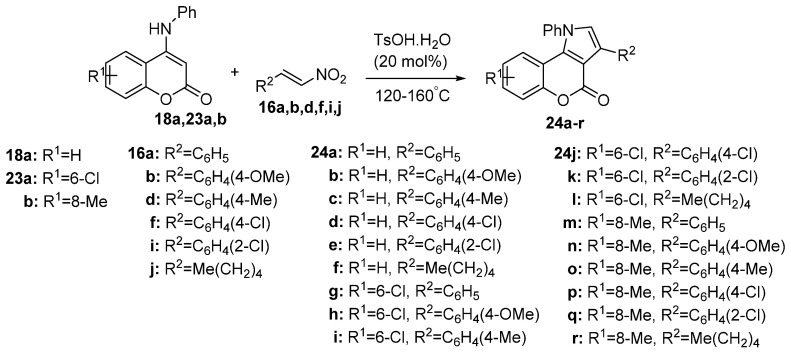
Synthesis of chromeno[4,3-*b*]pyrrol-4(1*H*)-ones **24a**–**f** from 4-*N*-phenylaminocoumarins **23a,b** and β-nitro alkenes **16** using TsOH.H_2_O as catalyst.

Padilha et al. used *p*-toluenesulfonic acid for the synthesis of chromeno[4,3-*b*]pyrrol-4(1*H*)-ones **24a**–**f** from 4-*N*-phenylaminocoumarins **23a**,**b** and β-nitro alkenes **16** under solvent-free conditions in 6–77% yield (Scheme 8). The proposed mechanism for the synthesis of compounds **24** was like the one proposed in Scheme 5 [76].

**Scheme 9:**
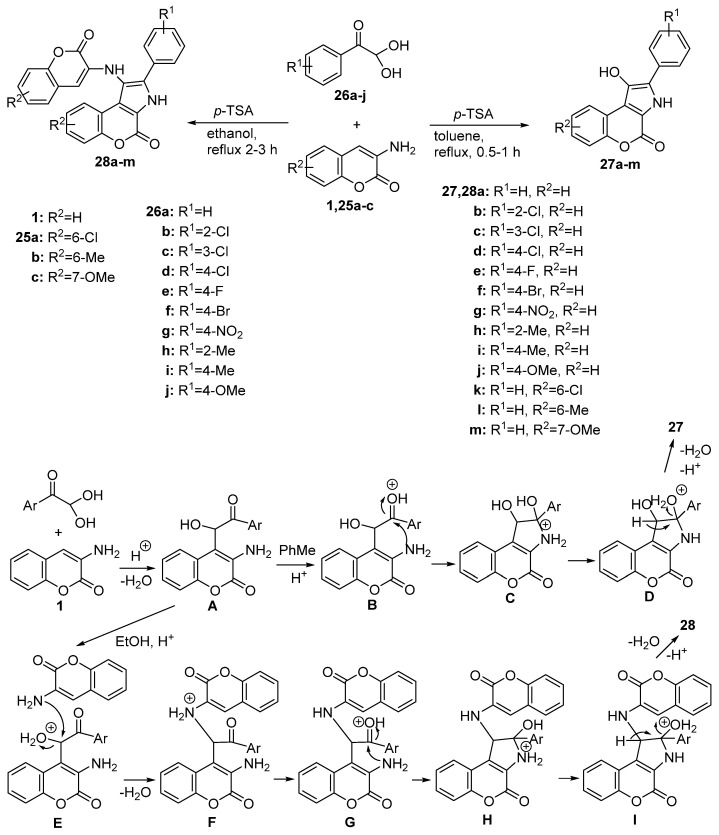
Synthesis of 2-aryl-1-hydroxychromeno[3,4-*b*]pyrrol-4(3*H*)-ones **27a**–**m** or 2-aryl-1-(2-oxo-2*H*-chromen-3-ylamino) chromeno[3,4-*b*]pyrrol-4(3*H*)-ones **28a**–**m** from 3-aminocoumarins and aryl glyoxal monohydrates.

Yang et al. reported the synthesis of chromeno[3,4-*b*]pyrrol-4(3*H*)-ones through the domino reaction of 3-aminocoumarins **1**, **25a**–**c** with aryl glyoxal monohydrates **26a**–**j** [77]. The reactions were catalyzed by *p*-toluenesulfonic acid (*p*-TSA). The solvents toluene or ethanol are important, as they affect the synthesis of the products 2-aryl-1-hydroxychromeno[3,4-*b*]pyrrol-4(3*H*)-ones **27a**–**m** or 2-aryl-1-(2-oxo-2*H*-chromen-3-ylamino) chromeno[3,4-*b*]pyrrol-4(3*H*)-ones **28a**–**m** in 54–78% or 75–92% yield, respectively (Scheme 9). In the proposed mechanistic scheme, the common intermediate **A** is formed by the nucleophilic attack of aminocoumarin on glyoxal. In the presence of toluene, intramolecular cyclization of **A** to **C** followed by dehydration of **D** led to the adduct **27**. In the presence of polar solvent, ethanol, protonation of the hydroxyl group of **A** afforded intermediate **E**. Nucleophilic attack of the amine group of 3-aminocoumarin furnished intermediate **F**, maybe for steric reasons. Intramolecular cyclization of **F** to **G** followed by dehydration of **I** resulted in the product **28**.

**Scheme 10:**
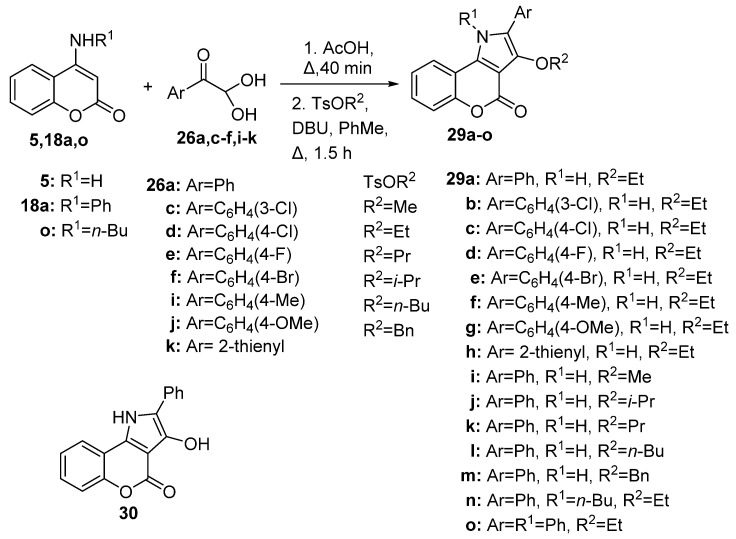
Synthesis of 3-alkoxychromeno[4,3*-b*]pyrrol-4(1*H*)-ones **29a**–**o** by the one-pot two-step reaction of 4-aminocoumarins with glyoxal monohydrates and *p*-toluene sulfonates.

The same group of Dr. Chen presented the synthesis of 3-alkoxychromeno[4,3-*b*]pyrrol-4(1*H*)-ones **29a**–**o** via the one-pot two-step reaction of 4-aminocoumarins **5**, **18a**,**o** with aryl glyoxal monohydrates **26** and *p*-toluene sulfonates (Scheme 10). In the first step of this reaction, an intermediate 3-hydroxychromeno[4,3-b]pyrrol-4(1*H*)-one, like **30**, was formed in an analogous process as described in Scheme 9, followed by nucleophilic substitution from the alkylating agent in the second step [78].

**Scheme 11:**
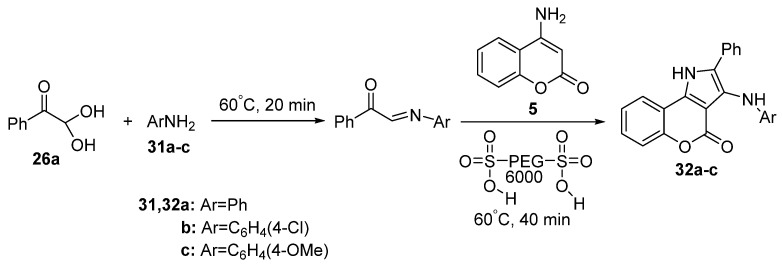
Synthesis of 3-arylaminochromeno[4,3-*b*]pyrrol-4(1*H*)-ones **32a**–**c** by the one-pot two-step reaction of phenyl glyoxal monohydrate (**26a**) with arylamines and 4-aminocoumarin (**5**).

Mukherjee et al. synthesized 3-arylaminochromeno[4,3-*b*]pyrrol-4(1*H*)-ones **32a**–**c** by a similar one-pot two-step reaction of phenyl glyoxal monohydrate (**26a**) with arylamine **31a**–**c** and 4-aminocoumarin (**13**) in the presence of Bronsted acid-surfactant reagent PEG-SO_3_H [53]. In the first step, the reaction of phenyl glyoxal with arylamine afforded imine derivative **A**. Michael addition of 4-aminocoumarin (**5**) to **A** in the second step, followed by cyclization and removal of water, resulted in the final product (Scheme 11). Compounds **32a**–**c** were found to be cell-permeable and exhibiting fluorescence “turn-off” in the presence of Cu^2+^ ions in fluorescent imaging of human cervical cancer (HeLa) cells.

**Scheme 12:**
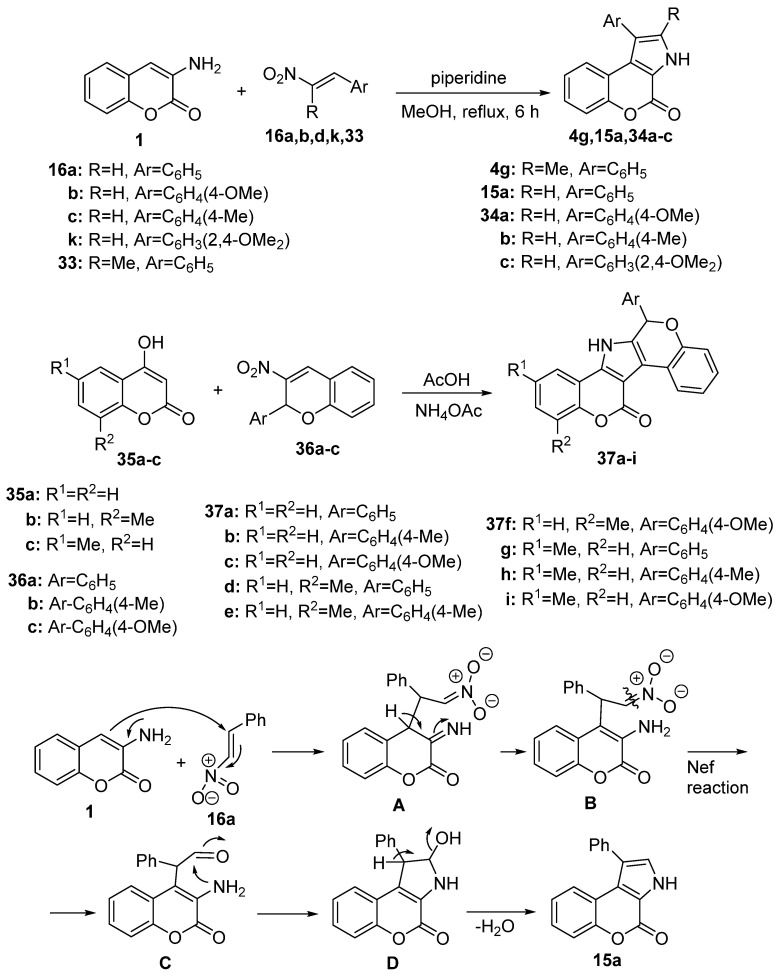
Synthesis of pyrrolo[2,3-*c*]coumarins **4g**, **15a**, **34a**–**c** and 1(*H*)[1]benzopyrano[3,4-*b*][1]benzopyrano[3′,4′-*b*]pyrrole-7(*H*)-ones **37a**–**i** from 3-aminocoumarin and 4-hydroxycoumarins, respectively, and β-nitrostyrenes.

Pandya et al. reported the synthesis of pyrrolo[2,3-*c*]coumarins derivatives **4g**, **15a**, **34a**–**c** in 51–57% yield from the reactions of 3-aminocoumarin (**1**) with β-nitrostyrenes **16a**,**b**,**d**,**k**, **33** in the presence of piperidine [79]. 1(*H*)[1]Benzopyrano[3,4-*b*][1]benzopyrano[3′,4′-b]pyrrole-7(*H*)-ones **37a**–**i** were synthesized also, in 51–56% yield from the reactions of 4-hydroxycoumarins **35a**–**c** with 3-nitro-2-aryl-2*H*-[1]benzopyrans **36a**–**c** in the presence of acetic acid and ammonium acetate (Scheme 12). For the mechanism, Michael addition of 3-aminocoumarin (**1**) to β-nitrostyrene (**16a**) gave intermediate **B** after tautomerization of **A**. Intermediate aldehyde **C** was formed next, according to Nef reaction conditions [80]. Intramolecular cyclization to **D** followed by elimination of water resulted in the pyrrolo[2,3-*c*]coumarin **15a**.

**Scheme 13:**
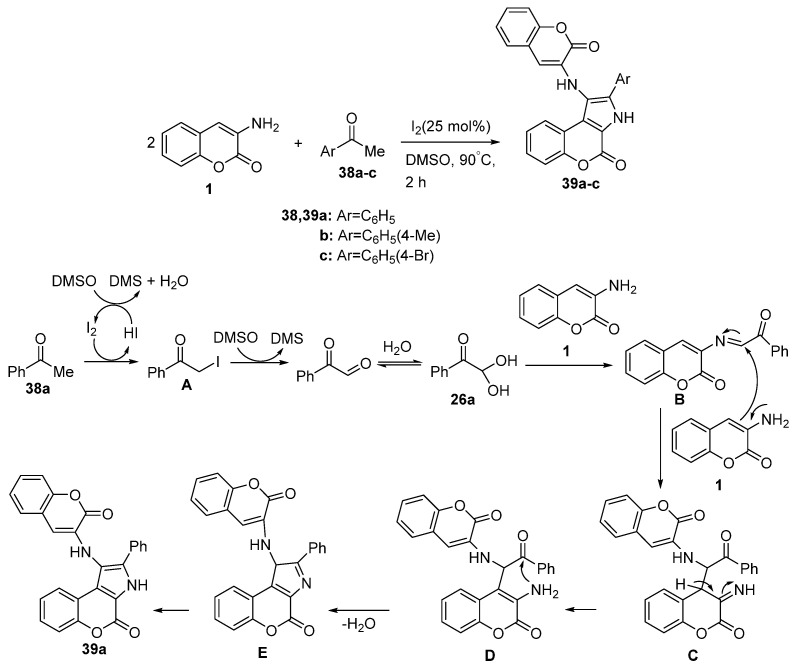
Synthesis of 2-aryl-1-((2-oxo-2*H*-chromen-3-yl)amino)chromeno[3,4-*b*]pyrrol-4(3*H*)-ones **39a**–**c**.

Recently, Islam et al. synthesized 2-aryl-1-((2-oxo-2*H*-chromen-3-yl)amino)chromeno[3,4-*b*]pyrrol-4(3*H*)-ones **39a**–**c** in 76–81% yield from the reaction of 3-aminocoumarin (**1**) with aryl methyl ketones **38a**–**c** in the presence of a catalytic amount of iodine in dimethyl sulfoxide (DMSO) [81]. In the proposed mechanism, phenacyl iodide (**A**) was generated by an electrophilic iodination, followed by oxidation leading to the phenyl glyoxal hydrate (**26a**). Reaction of the latter with 3-aminocoumarin (**1**) gave imine **B**. Michael addition of a second molecule of **1** to **B** resulted in the intermediate **D** through the tautomerization of imine **C**. By cyclization of **D**, followed by tautomerization of **E**, the final product **39a** was formed (Scheme 13). 

#### 2.1.2. Synthesis from Other Coumarin Derivatives

**Scheme 14:**
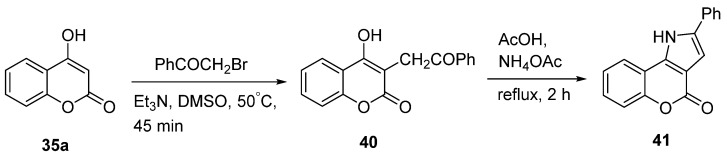
Synthesis of 2-phenyl-4*H*-pyrro1[3,2-*c*][1]benzopyran-4-one (**41**).

Trkovnic et al. reported the synthesis of 2-phenyl-4*H*-pyrro1o[3,2-*c*][1]benzopyran-4-one (**41**) in 83% yield by the treatment of 4-hydroxy-3-phenacylcoumarin (**40**) with acetic acid in the presence of ammonium acetate [82]. Compound **40** was synthesized in 59% yield by the reaction of 4-hydroxycoumarin (**35a**) with phenacyl bromide (Scheme 14).

**Scheme 15:**
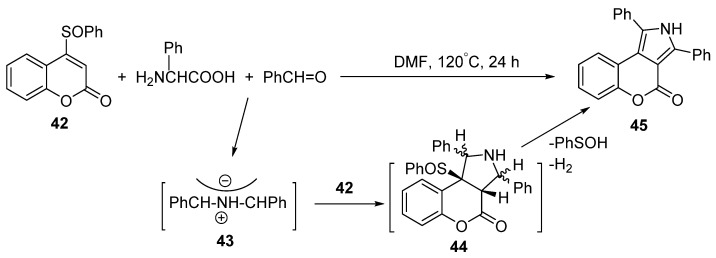
Synthesis of 1,3-diphenylpyrrolo[3,4-*c*]chromene-4-one (**45**).

Grigg and Vipond synthesized the 1,3-diphenylpyrrolo[3,4-*c*]chromene-4-one (**45**) in 51% yield by the 1,3-dipolar cycloaddition reaction of nitrile imine **43**, prepared in situ from phenylglycine and benzaldehyde [83]. Thermal elimination of phenylsulfenic acid from the intermediate dihydropyrrole derivative **44** followed by oxidation in the reaction conditions resulted in the final product **45** (Scheme 15).

**Scheme 16:**
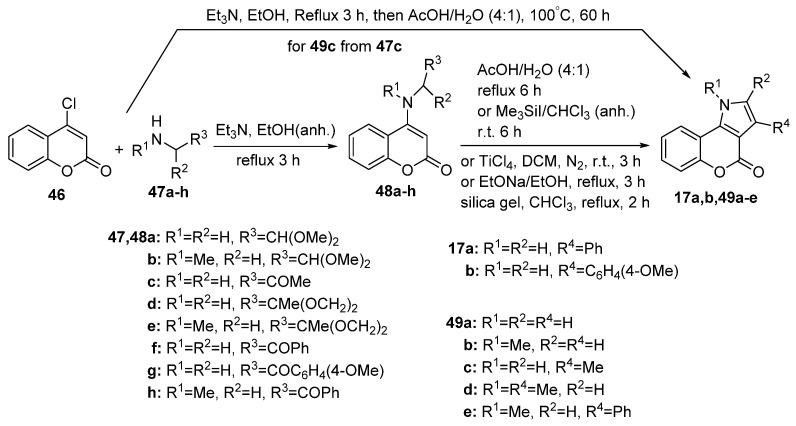
Synthesis of [1] benzopyrano[4,3-*b*]pyrrol-4(1*H*)-ones **17a**,**b**, **49a**–**e** from 4-chlorocoumarin (**46**).

Alberola et al. prepared [1] benzopyrano[4,3-*b*]pyrrol-4(1*H*)-ones **17a**,**b**, **49a**–**e** from 4-acylamino- or 4-ketalaminocoumarin derivatives **48a**–**h** under different conditions using acetic acid/water, or Me_3_SiI in chloroform or TiCl_4_ in dichloromethane (DCM) or EtONa in ethanol or silica gel in chloroform [84]. Compounds **49a**–**e** were prepared in high yields of 71–92% through the method with acetic acid/water. TiCl_4_ in DCM resulted in the synthesis of **49b**,**d** in yields of 88% and 92%, respectively. Compounds **17a**,**b** were synthesized from **48f**,**g** under treatment with EtONa in ethanol in 75% and 89% yields, respectively. Compound **49e** was prepared from **48h** in silica gel and chloroform in 89% yield. One-pot reaction of **46** with **47c** afforded compound **49c** in 80% yield (Scheme 16). 4-Acylamino- and 4-ketalaminocoumarin derivatives **48a**–**h** were synthesized from the reaction of 4-chlorocoumarin (**46**) with acylamine chlorohydrates or ketalamines **47a**–**h** under treatment with Et_3_N in refluxing ethanol. 

**Scheme 17:**
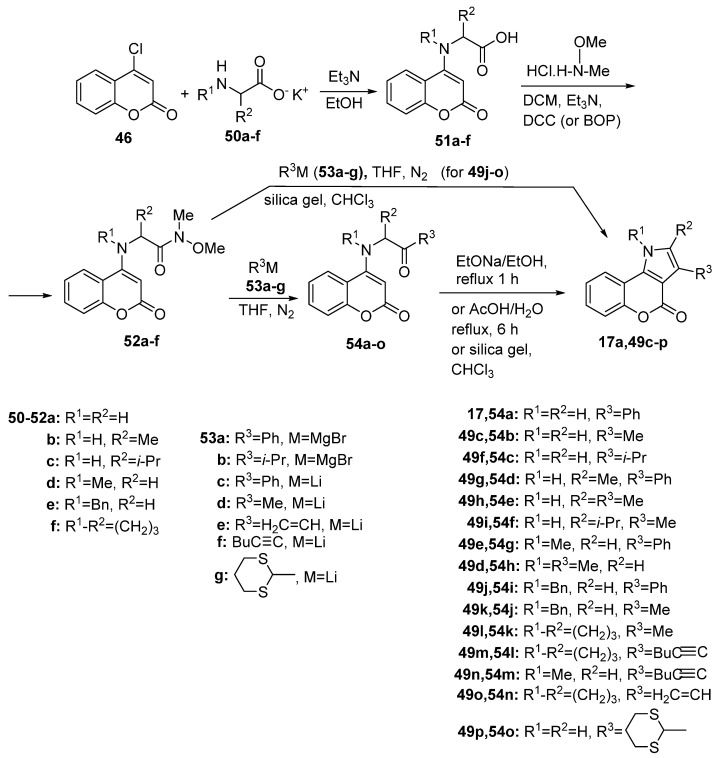
Synthesis of [1] benzopyrano[4,3-*b*]pyrrol-4(1*H*)-ones **17a**, **49c**–**p** from *N(α)*-(2-oxo-2*H*-1-benzopyran-4-yl) Weinreb α-aminoamides.

The same group of Alberola et al. reported the synthesis of [1] benzopyrano[4,3-*b*]pyrrol-4(1*H*)-ones **17a**, **49c**–**p** from the Weinreb α-aminoamides **52a**–**f** [85]. The *N(α)*-(2-oxo-2*H*-1-benzopyran-4-yl) Weinreb α-aminoamides (**52a**–**f**) were prepared in 73–87% yield from the α-aminoacid derivatives **51a**–**f** (generated from the reaction of 4-chlorocoumarin (**46**) with α-aminoacid potassium salt) under treatment with *N,O*-dimethyl hydroxylamine. Weinreb amides reacted with organomagnesium or organolithium compounds **53a**–**g** to give 4-ketonylaminocoumarins **54a**–**h**,**o** in 51–92% yield (Scheme 17). Cyclization of the latter under different conditions, EtONa in ethanol (for **54a**–**f**,**o**), acetic acid/water (for **54g**) or evaporation with silica gel in chloroform (for **54h**), furnished the fused pyrrolocoumarins **17a**, **49c**–**i**,**p** in 69–92% yield. The reactions of Weinreb amides with organolithium compounds in silica gel and chloroform, in the case of the expected 4-ketonylaminocoumarins **54i**–**n**, led through a one-pot process to the cyclization products **49j**–**o** in 15–81% yield without the isolation of the intermediates.

**Scheme 18:**
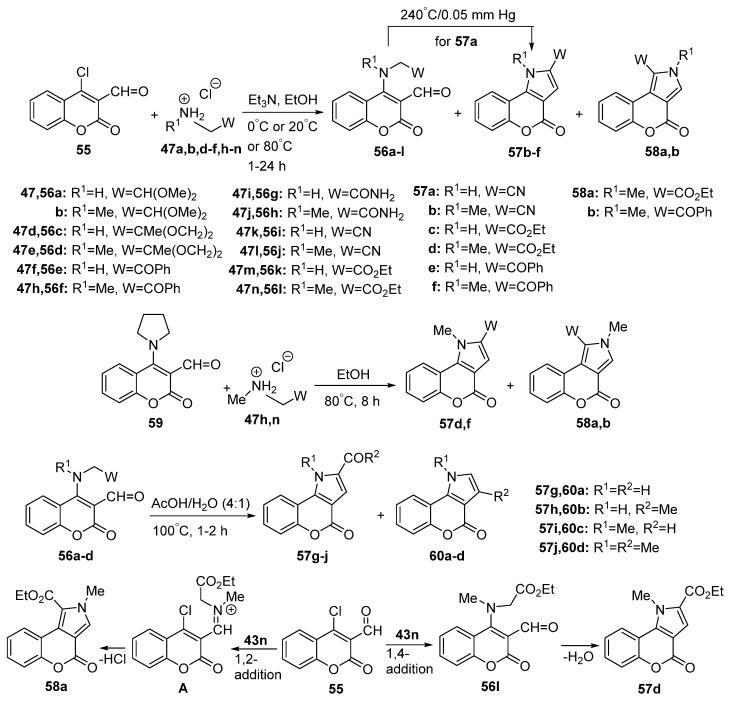
Synthesis of [1] benzopyrano[4,3-*b*]pyrrol-4(1*H*)-ones **57a**–**f** from 4-chloro-3-formylcoumarin.

The same group of researchers presented the synthesis of [1] benzopyrano[4,3-*b*]pyrrol-4(1*H*)-ones **57a**–**f** by the Fischer–Fink reaction from 4-chloro-3-formylcoumarin (**55**) and a-amino derivatives as hydrochloric acid salts **47f**,**h**–**n** [86]. Reaction of **55** with **47a**,**b**,**d**–**f**,**h**–**n** in ethanol in the presence of Et_3_N (three equivalents) mainly at 0 °C (60 °C for **56g** or 20 °C for **56a**–**d**, without Et_3_N in chloroform) for 1–12 h resulted in the synthesis of 3-amino-4-formylcoumarins **56a**–**l** in 73–95% yield (Scheme 18). The same reaction at 80 °C (or 20 °C for 24 h for **57b**) for 2–12 h afforded the expected pyrrolocoumarins **57b**–**f** in 71–90% yield. Sublimation of **56i** at 240 °C/0.05 mm Hg furnished **57a** in 25% yield. The similar reaction of **55** with **47n** in the presence of one equivalent of Et_3_N for 12 h led to the synthesis of **57d** in 33% yield and to the isolation of 1-ethoxycarbonyl-2-methylchromeno[3,4-*c*]pyrrol-4(2*H*)-one (**58a**) in 51% yield. The reaction of 3-formyl-4-pyrrolidinylocoumarin (**59**) with **47n** without the presence of base for 8 h resulted in the adducts **57d** (29%) and **58a** (58%). From the reaction of **59** with **47h** without base for 8 h, products **57f** (4%) and 1-benzoyl-2-methylchromeno[3,4-*c*]pyrrol-4(2*H*)-one (**58b**) (79%) were obtained. The cleavage of acetals or ketals **56a**–**d** in the presence of acetic acid-water resulted in the fused pyrroles **57g**–**j** and **60a**–**d** in 30–87% yield under cyclization and elimination of water or formic acid, respectively. A possible mechanistic process for the formation of 4-amino-3-formylocoumarin **56l** occurred through 1,4-addition of **47n** to **55**, which after elimination of water resulted in the pyrrolo[4,3-*b*]coumarin **57d**. The less possible 1,2-addition of **47n** to **55**, for the formation of intermediate **A**, followed by elimination of HCl could produce pyrrolo[3,4-*c*]coumarin **58a**. 

**Scheme 19:**
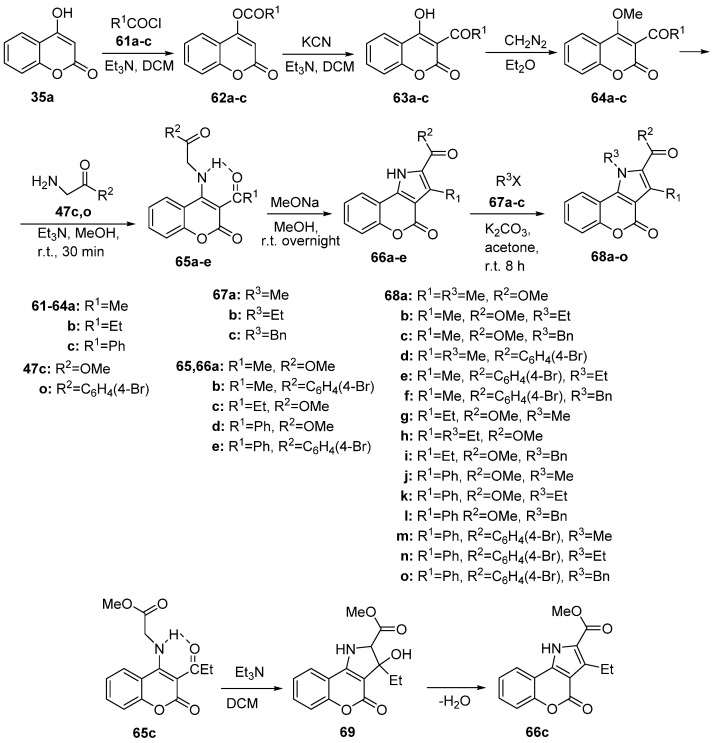
Synthesis of [1] benzopyrano[4,3-*b*]pyrrol-4(1*H*)-ones **66a**–**e** from 4-hydroxycoumarin (**35a**).

Liao et al. synthesized [1] benzopyrano[4,3-*b*]pyrrol-4(1*H*)-ones **66a**–**e** from 4-hydroxycoumarin (**35a**) in 30–40% yield through six steps (acylation, isomerization, protection of hydroxyl group, nucleophilic substitution) and the cyclization of enamines **65a**–**e** followed by dehydration [87]. For the mechanism of the cyclization reaction, the intermediate hydroxy compound **69** was isolated during the treatment of enamine **65c** with base (Et_3_N) (Scheme 19). Hydroxy derivative **69** resulted in the final product **66c** under elimination of water. N-Alkylation of **66a**–**e** with halides **67a**–**c** in the presence of K_2_CO_3_ at room temperature afforded the trisubstituted fused pyrrolocoumarins **68a**–**o**.

**Scheme 20:**
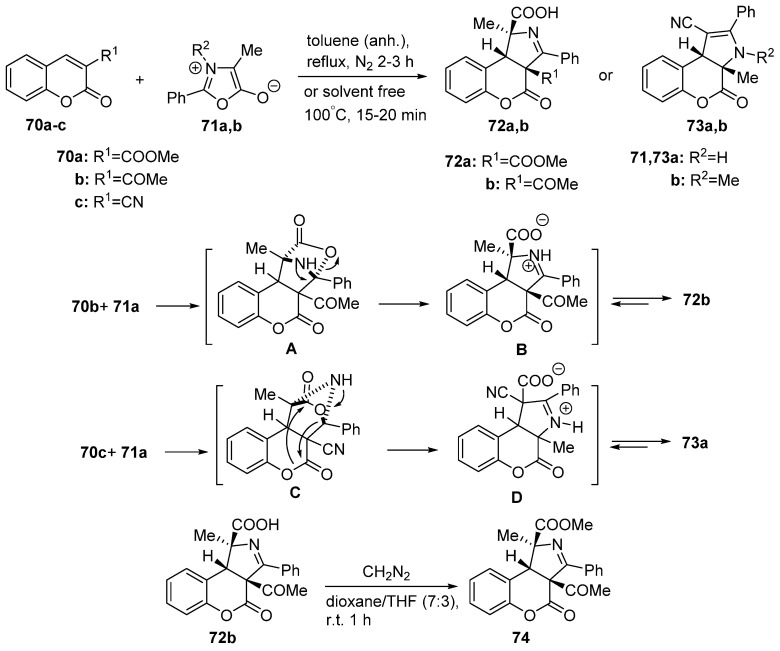
Synthesis of fused dihydropyrrolo[3,4-*c*]coumarin derivatives **72a**,**b**, **73a**,**b**, **74**.

Cordaro et al. examined the 1,3-dipolar cycloaddition reaction of unsymmetrically *N*-unsubstituted and *N*-substituted 1,3-oxazolium-5-olates **71a**,**b** with 3-substituted coumarins **70a**–**c**, bearing electron-withdrawing group [88]. The products **72a**,**b** were obtained with both reaction conditions in solution (69–77% yield) or without solvent (80–89% yield), while products **73a**,**b** were obtained only in solution in 40% or 15% yield, respectively. In the proposed mechanism of oxazolone cycloaddition reaction, the formation of products **72a**,**b** could be explained through *exo*-diastereoisomer **A** and the intermediate **B** leading to stable α-aminoacids (Scheme 20). *Endo*-diastereoisomer **C** with translactonization to **D** and elimination of CO_2_ is responsible for the formation of products **73a**,**b**.

**Scheme 21:**
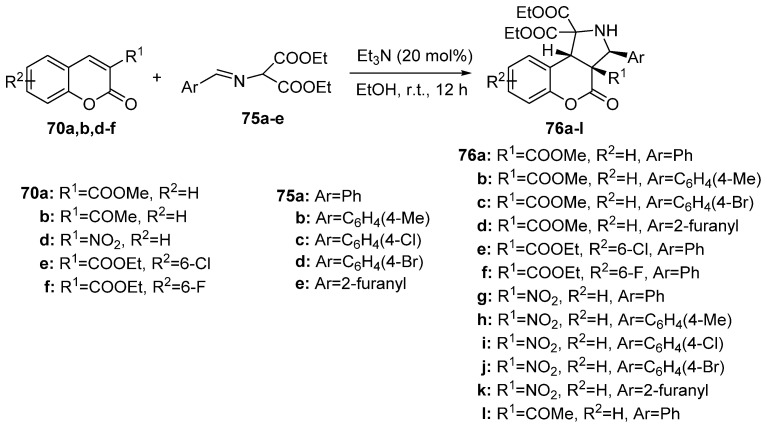
Synthesis of benzopyran [3,4-*c*]pyrrolidines **76a**–**l**.

Fan et al. prepared benzopyran [3,4-*c*]pyrrolidines **76a**–**l** in 73–97% yield by the triethylamine-catalyzed 1,3-dipolar cycloaddition reaction of azomethine ylides with 3-substituted coumarins [89]. Azomethine ylides were formed in situ from α-amino malonate imines **75a**–**e**. As they have been examined by X-ray analysis of **76e**, the products have the *cis* stereochemistry (Scheme 21).

**Scheme 22:**
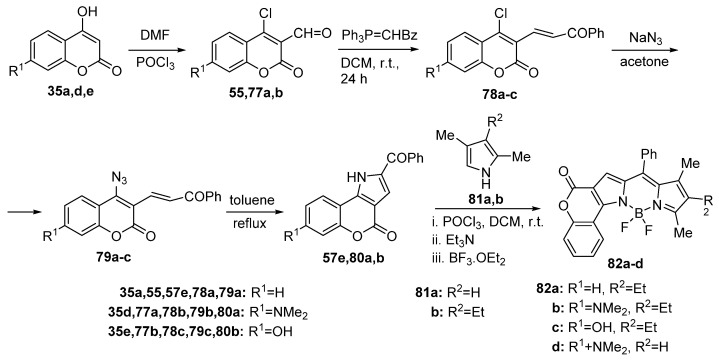
Synthesis of 2-benzoylchromeno[4,3-*b*]pyrrol-4-ones **57e**, **80a**,**b** and coumarin-fused BODIPY dyes **82a**–**d**.

Bochkov et al. [90] synthesized 2-benzoylchromeno[4,3-*b*]pyrrol-4-ones **57e**, **80a**,**b** starting from 4-hydroxycoumarins **35a**,**d**,**e** in four steps through 4-chloro-3-formylcoumarins **55**, **77a**,**b** in good yields, avoiding the formation of byproducts [85]. Wittig reaction of **55**, **77a**,**b** to give **78a**–**c**, followed by azide formation of **79a**–**c**, and thermal cyclization, furnished compounds **57e**, **80a**,**b** (Scheme 22). Reactions of the latter with pyrroles **81a**,**b** resulted in 4,4-difluoro-4-bora-3*a*,4*a*-diaza-*s*-indacene (BODIPY) dyes **82a**–**d** presenting large Stokes shifts and good fluorescence quantum yields, especially for **82b**,**d**.

**Scheme 23:**
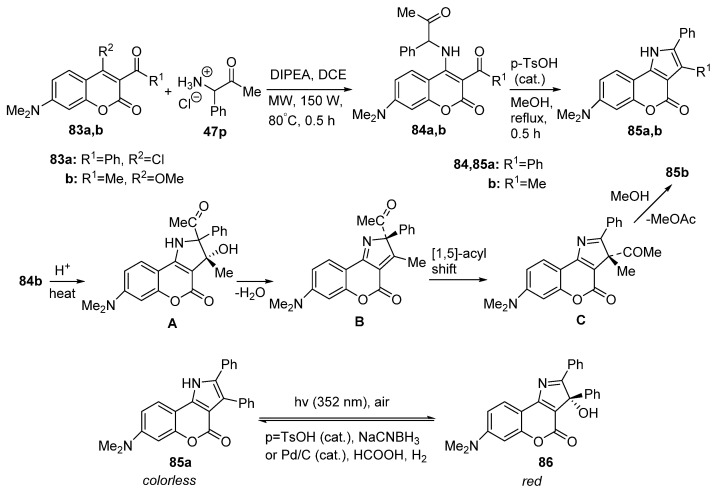
Synthesis of 7-*N,N*-dimethylaminochromeno[4,3-*b*]pyrrol-4-ones **85a**,**b**.

Lin and Yang prepared 7-*N,N*-dimethylaminochromeno[4,3-*b*]pyrrol-4-ones **85a**,**b** in excellent yields to study their photochemical and redox switching properties [91]. The synthesis was performed by the reaction of 3-acylcoumarins **83a**,**b** with α-aminoketone hydrochloric salt **47p** under microwave irradiation in the presence of diisopropyl ethyl amine (Scheme 23). The enamine derivatives **84a**,**b** under reflux in the presence of a catalytic amount of *p*-toluenesulfonic acid afforded the final product. In the proposed mechanism, the acid-catalyzed cyclization of **84b** furnished the intermediate **A**, which upon 1,4-elimination of water produced **B**. [1,5]-acyl migration afforded intermediate **C**. Methanolysis of **C** resulted in the fused pyrrolocoumarin **85b**. The fused pyrrolocoumarins were found to be sensitive to UV light upon changing the light under this wavelength. The product **86** of the transformation was stable, and it has been isolated.

**Scheme 24:**
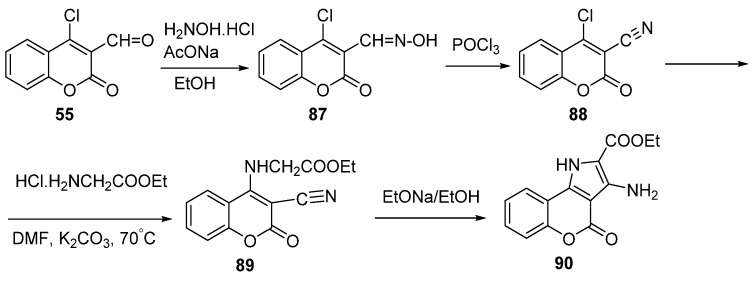
Synthesis of ethyl 3-amino-4-oxo-1,4-dihydrochromeno[4,3-*b*]pyrrole-2-carboxylate (**90**).

Kamal El-Dean et al. synthesized ethyl 3-amino-4-oxo-1,4-dihydrochromeno[4,3-*b*]pyrrole-2-carboxylate (**90**) from 4-chloro-3-cyanocoumarin (**88**) through the adduct with glycine ethyl ester (**47m**) followed by cyclization (Scheme 24). This new compound presented low antifungal activity as tested against *Candida albicans* and *Geotrichum candidum* [92].

**Scheme 25:**
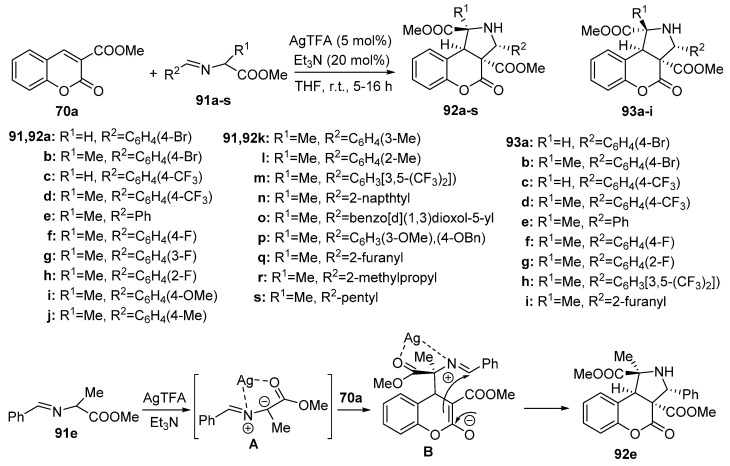
Synthesis of benzopyrano[3,4-*c*]pyrrolidines via Ag(I)-catalyzed 1,3-dipolar cycloaddition reaction.

Potowski et al. synthesized benzopyrano[3,4-*c*]pyrrolidines via AgTFA-catalyzed 1,3-dipolar cycloaddition reaction of coumarin **70a** with azomethine ylides formed from α-imino esters **91a**–**s** [93]. The *trans* diastereoisomers **92a**–**s** were obtained in 37–72% yield, while the *cis* diastereoisomers **93a**–**i** were isolated in only 9–34% yield. The configuration of the trans-product **92f** was confirmed by X-ray crystallographic analysis. A stepwise mechanism has been proposed for these reactions (Scheme 25). In particular, a-imino ester **91e** was coordinated to Ag(I) ion, and deprotonation from Et_3_N resulted in the intermediate azomethine ylide **A**. Nucleophilic attack of the latter to 4-C of coumarin furnished **B**, which upon cyclization afforded the *trans*-product **92e**.

**Scheme 26:**
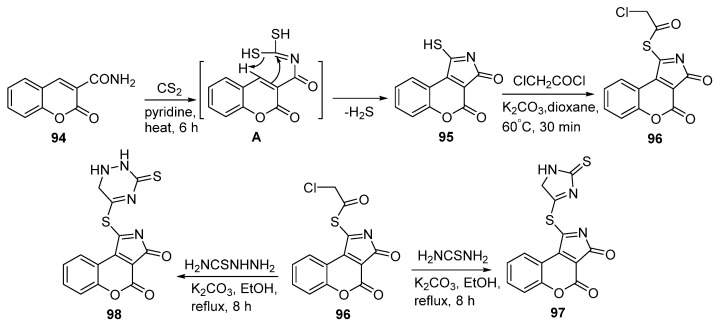
Synthesis of 1-mercapto- or 1-thiochromeno[3,4-*c*[pyrrol-3,4-dione derivatives **95**–**98**.

El Azab and Elkanzi presented the synthesis and biological evaluation of 1-mercapto- or 1-thiochromeno[3,4-*c*[pyrrol-3,4-dione derivatives **95**–**98** as antimicrobial agents [94]. The reaction of coumarin-3-ylcarboxamide (**94**) with CS_2_ led via the intermediate **A** to 1-mercaptopyrrolo[3,4-*c*]chromene-3,4-dione (**95**) in 85% yield (Scheme 26). Derivative **94** was the key compound for the synthesis of chloroacetylthio-derivative **96**, the heterocyclic derivatives **97**, **98** and other *N*-heterocycle derivatives. All the new compounds were tested for their in vitro antibacterial activity against two Gram-positive species, *Bacillus subtilis* RCMB 010067 and *Staphylococcus aureus* RCMB 0100010, and two Gram-negative species, *Pseudomonas aeuroginosa* RCMB 010043 and *Escherichia coli* RCMB 010052. Compound **98** presented high activity, greater against Gram-positive bacteria than the Gram-negative ones. The compounds were also evaluated for their in vitro antifungal activity against two fungal species, *Aspergillus fumigatus* RCMB 02568 and *Candida albicans* RCMB 05036. Compound **96** demonstrated high antifungal activity, while compound **98** presented moderate activity, and compound **97** had low activity. 

**Scheme 27:**
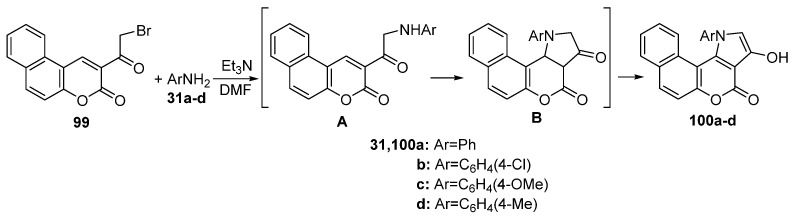
Synthesis of 3-hydroxy-1-arylbenzo[5,6]chromeno[4,3-*b*]pyrrol-4(1H)-ones **100a**–**d**.

Mohamed and Elbialy synthesized benzo[5,6]chromeno[4,3-*b*]pyrrol-4(1*H*)-one derivatives **100a**–**d** under treatment of 3-bromoacetylcoumarin derivative **99** with amines **31a**–**d** [38]. Nucleophilic substitution of bromine in **99** from amine afforded the intermediate **A**, which upon intramolecular Michael addition furnished intermediate **B**. The latter under autoxidation and enolization resulted in the product **100** (Scheme 27). New compounds **100a**–**d** were examined in vitro against HepG2 (hepatoma cell line) and MCF7 (human breast adenocarcinoma cell line) cell lines, exhibiting moderate cytotoxic activity.

**Scheme 28:**
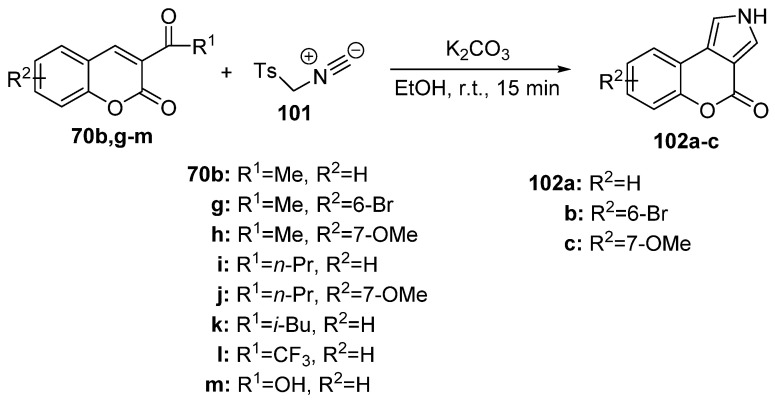
Synthesis of chromeno[3,4-*c*]pyrroles **102a**–**c** by [3 + 2] cycloaddition reaction of TosMIC with coumarins.

Shaabani et al. reported the regioselective reaction of tosylmethyl isocyanide (TosMIC) (**101**) with the double bond of 3-carbonyl coumarin derivatives for the synthesis of chromeno[3,4-*c*]pyrroles **102a**–**c** (Scheme 28). The above reaction is a Van Leusen type [3 + 2] cycloaddition reaction of TosMIC with the 3,4-double bond of coumarin [95]. 

**Scheme 29:**
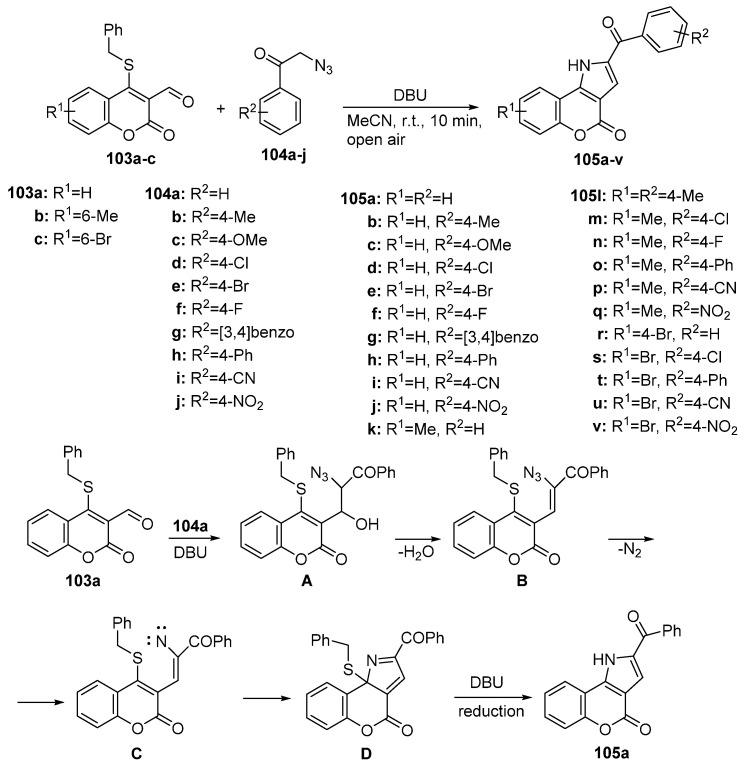
Synthesis of 2-benzoyl chromeno[4,3-*b*]pyrrol-4(1*H*)-ones **105a**–**v**.

Recently, Borkotoki et al. reported the synthesis of 2-benzoyl chromeno[4,3-*b*]pyrrol-4(1*H*)-ones **105a**–**v** in 67–84% yield through the DBU-mediated coupling of 3-formylocoumarin derivatives **103a**–**c** with phenacyl azides **104a**–**j** [96]. As a possible mechanistic interpretation, the DBU-mediated coupling of azide **104a** with coumarin-3-carbaldehyde **103a** afforded the intermediate azidoalcohol **A** (Scheme 29). Dehydration of **A** furnished **B**. Elimination of nitrogen produced nitrene **C**, which upon intramolecular cyclization led to intermediate **D**. Reduction by DBU in acetonitrile resulted in the final product **105a**.

#### 2.1.3. Synthesis through MCR Reactions

**Scheme 30:**
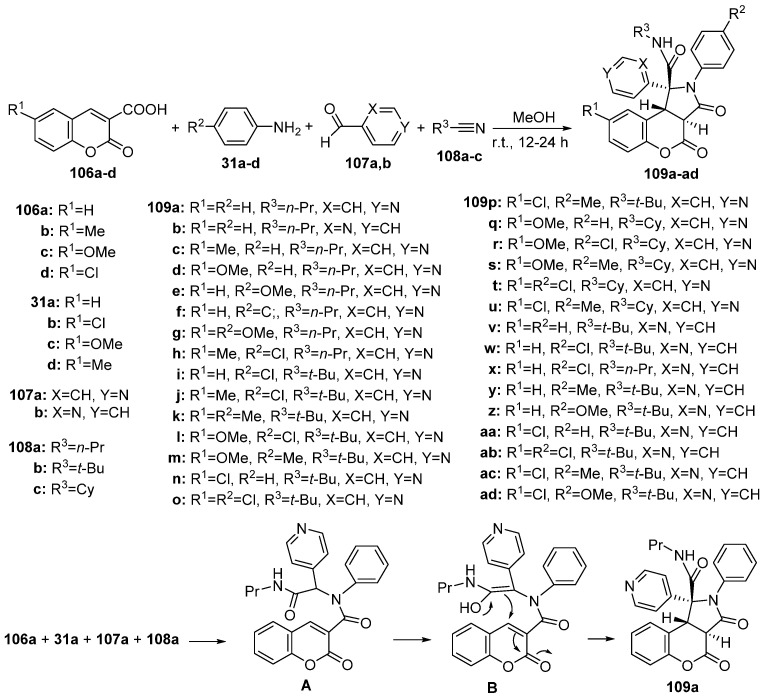
Synthesis of chromeno[3,4-*c*]pyrrol-2,4-dione derivatives **109a**–**ad**.

The use of multicomponent reaction (MCR) was reported for the first time by Che et al. for the synthesis of fused pyrrolocoumarins. Chromeno[3,4-*c*]pyrrol-2,4-dione derivatives **109a**–**ad** were synthesized in 45–82% yield during the one-pot process via the Ugi-4CR followed by intramolecular Michael addition of coumarin-3-carboxylic acid **106a**–**d**, anilines **31a**–**d**, nitriles **108a**–**c**, and aldehydes **107a**,**b** [97]. The acylaminoamide **A** was formed by the Ugi reaction of **106a**, **31a**, **107a**, and **108a**, and after enolization, the intermediate **B** was cyclized to afford the final product **109a** (Scheme 30).

**Scheme 31:**
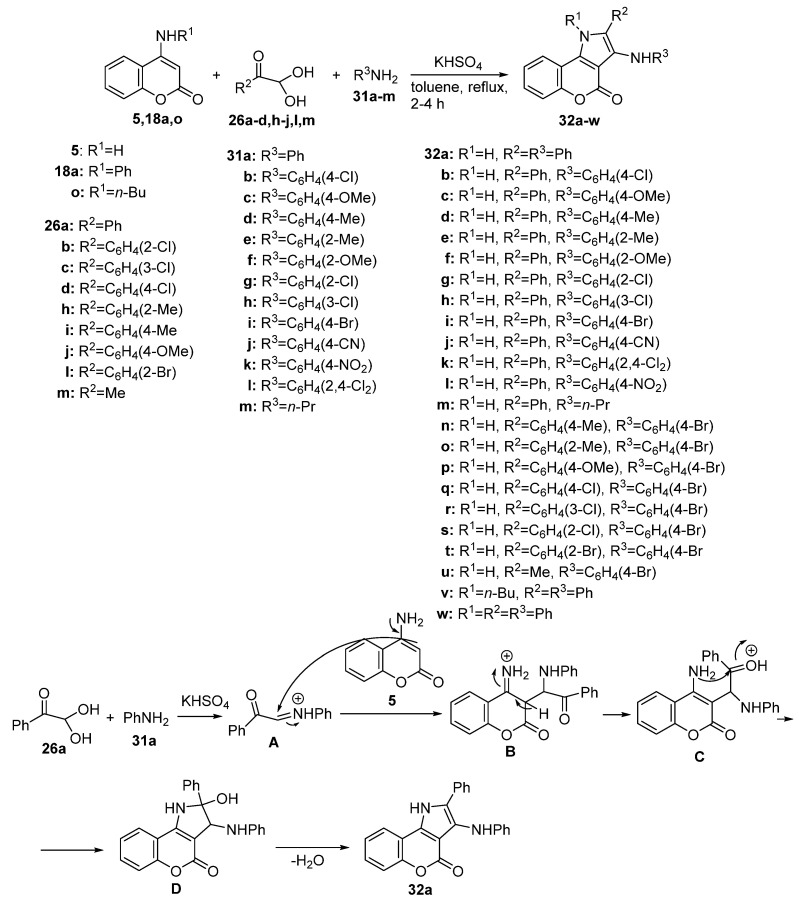
Synthesis of fused pyrrolocoumarins **32a**–**w** via MCR.

Chen et al. reported the use of aryl glyoxals in the MCR synthesis of fused pyrrolocoumarins [98]. Aryl glyoxals have been used extensively for the synthesis of fused pyrrolocoumarins [61]. In this work, trisubstituted chromeno[4,3-*b*]pyrrol-4(H)-ones **32a**–**w** have been synthesized in 34–93% yield via one-pot three-component reaction of 4-aminocoumarins, aryl glyoxal monohydrates, and aryl amines **31a**–**m** (Scheme 31). In the proposed mechanism, an initial condensation in the presence of KHSO_4_ of phenyl glyoxal monohydrate (**26a**) with aniline (**31a**) furnished the intermediate imine **A**. The reaction of **A** with 4-aminocoumarin (**5**) provided the intermediate **B**. Intramolecular cyclization of **C**, tautomer of intermediate **B**, afforded dihydropyrrolocoumarin **D**, which upon dehydration resulted in the final product **32a**.

**Scheme 32:**
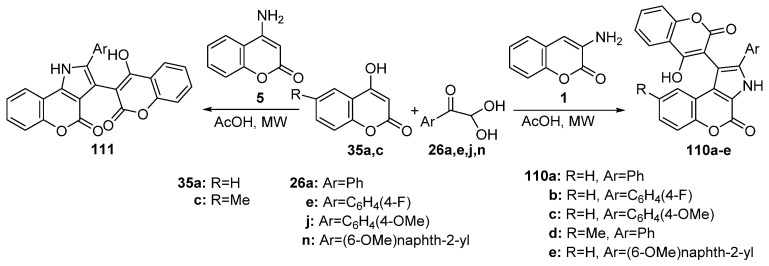
Synthesis of coumarin-3-yl-substituted fused pyrrolocoumarin derivatives **110a**–**e** and **111**.

Mishra et al. reported the synthesis of coumarin-3-yl-substituted fused pyrrolocoumarin derivatives **110a**–**e** or **111** in 90–95% yield by the three-component reaction of 4-hydroxycoumarin, aryl glyoxal monohydrates, and 3-aminocoumarin (**1**) or 4-aminocoumarin (**5**), respectively, under microwave irradiation (Scheme 32). Discrete Fourier transform (DFT) calculations proved that both ketone and aldehyde group of arylglyoxal participate in the formation of the final products [99].

**Scheme 33:**
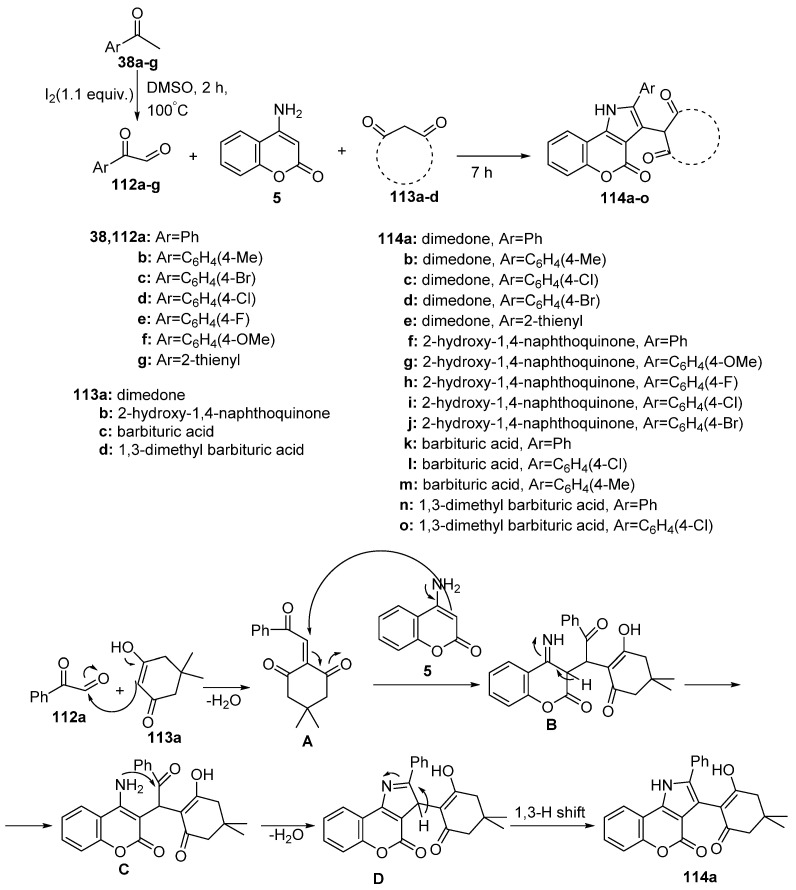
Synthesis of 2,3-disubstituted chromeno[4,3-*b*]pyrrol-4(H)-ones **112a**–**o** via iodine-catalyzed multicomponent reaction.

Yahyavi et al. synthesized 2,3-disubstituted chromeno[4,3-*b*]pyrrol-4(H)-ones **114a**–**o** in 73–89% yield via the iodine-catalyzed one-pot MCR of aryl glyoxals **112a**–**g**, prepared in situ by the Kornblum oxidation of acetophenones **38a**–**g**, active methylene compounds **113a**–**d** and 4-aminocoumarin (**5**) [100]. In the proposed mechanism, the initially formed phenyl glyoxal (**112a**) was condensed in a Knoevenagel reaction assisted by iodine with dimedone (**113a**) to give intermediate **A**. Michael addition of 4-aminocoumarin (**5**) to **A** afforded imine **B**, which upon tautomerization to **C** and cyclization with elimination of water furnished intermediate **D**. 1,3-H Shift of the latter resulted in the final product **114a** (Scheme 33).

**Scheme 34:**
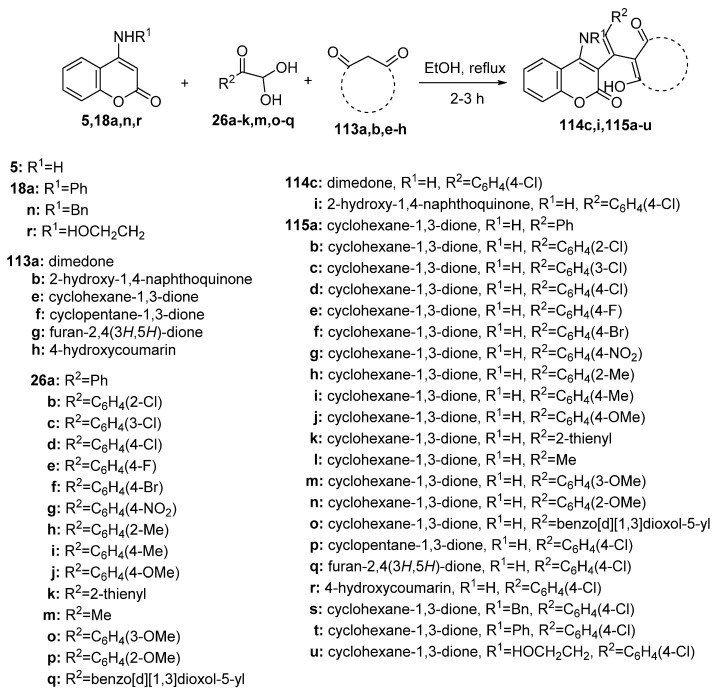
Synthesis of substituted chromeno[4,3-*b*]pyrrol-4(H)-ones **114c**,**i** and **115a**–**u** via multicomponent reaction under catalyst-free conditions.

A similar MCR without catalyst in refluxing ethanol was reported by Yang et al. for the synthesis of chromeno[4,3-*b*]pyrrol-4(H)-one derivatives [101]. The one-pot reaction of 4-aminocoumarins, aryl glyoxal monohydrates and 1,3-dicarbonyl compounds resulted in the synthesis of substituted chromeno[4,3-*b*]pyrrolo-4(H)-ones **114c**,**i** and **115a**–**u** in 35–89% yield (Scheme 34). The proposed mechanism resembles the above mechanistic scheme.

**Scheme 35:**
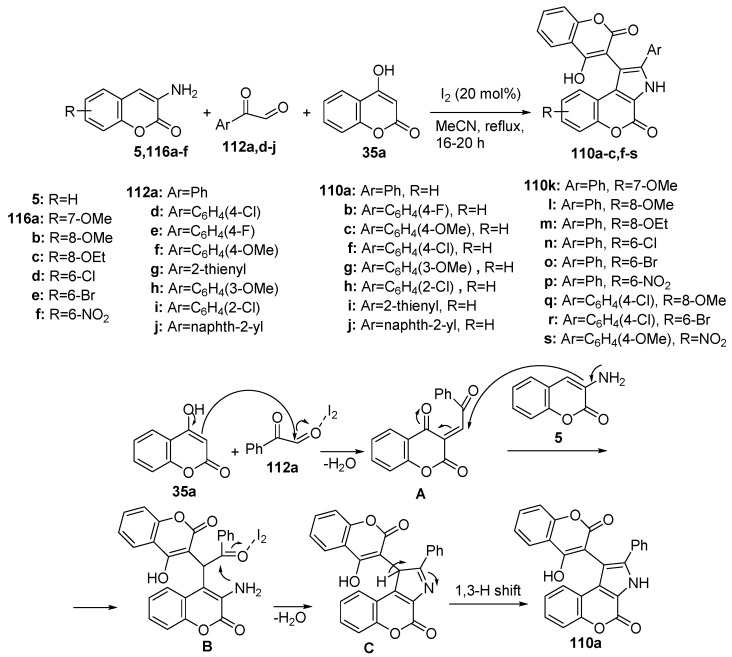
Synthesis of pyrrolo[2,3-*c*]coumarin derivatives **110** by iodine-catalyzed three-component reaction.

Belal and Khan reported analogous, iodine-catalyzed, multicomponent reactions for the synthesis of pyrrolo[2,3-*c*]coumarin derivatives **110** in 78–86% yield from 3-aminocoumarin, aryl glyoxal **112**, and 4-hydroxycoumarin (**35a**) [102]. The mechanism for this one-pot three-component iodine-catalyzed reaction is like the one presented in Scheme 33 (Scheme 35). 

**Scheme 36:**
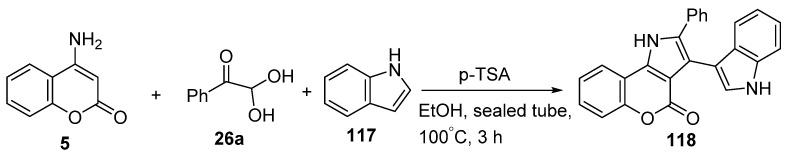
Synthesis of 3-(indol-3-yl)substituted chromeno[4,3-*b*]pyrrol-4(*H*)-ones.

Chen et al. also reported the synthesis of 3-(indol-3-yl)substituted chromeno[4,3-*b*]pyrrol-4(H)-ones, like **118** (Scheme 36), via a three-component reaction of aminocoumarins, aryl glyoxal monohydrates and indoles [103].

**Scheme 37:**
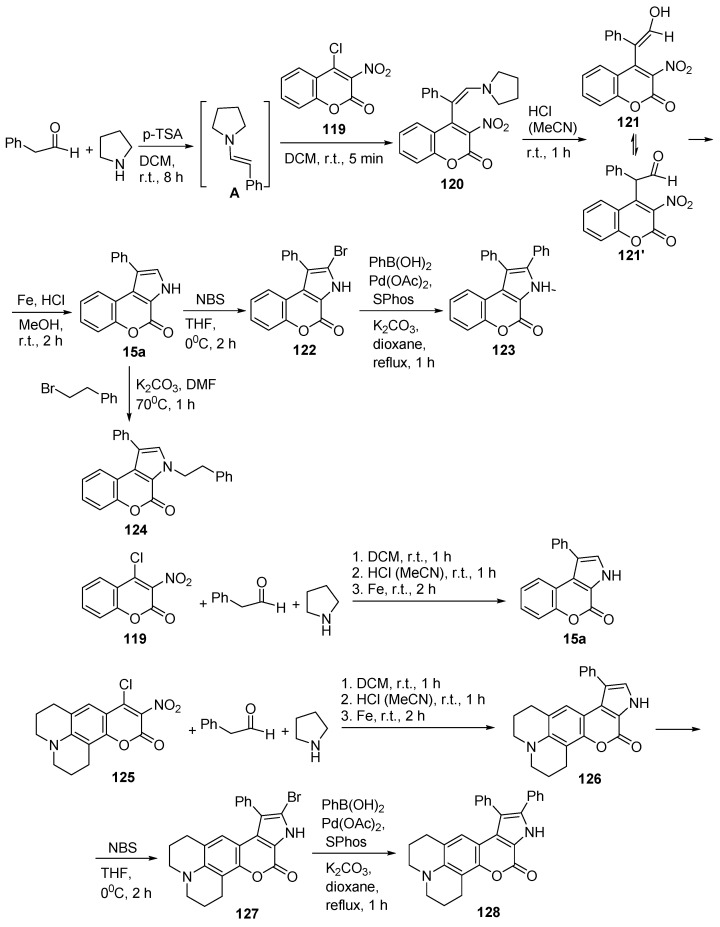
One-pot synthesis of 1-phenylchromeno[3,4-*b*]pyrrol-4(3*H*)-one (**15a**).

Wu et al. presented the one-pot synthesis of 1-phenylchromeno[3,4-*b*]pyrrol-4(3*H*)-one (**15a**) through the three-component reaction of phenylacetaldehyde, pyrrolidine, and 4-chloro-3-nitrocoumarin (**119**) in three steps and 78% yield [104]. Similarly, they also synthesized the chromeno[3,4-*b*]pyrrol-4(3*H*)-one derivatives **122**-**124** and **126**-**128** from compound **125** (Scheme 37). They utilized this method for the synthesis of ningalin B. The key step of this reaction was the coupling between the intermediate 1-styrylpyrrolidine **A** and 4-chloro-3-nitrocoumarin (**119**). Diphenyl derivative **128** exhibits electrochromic properties. 

**Scheme 38:**
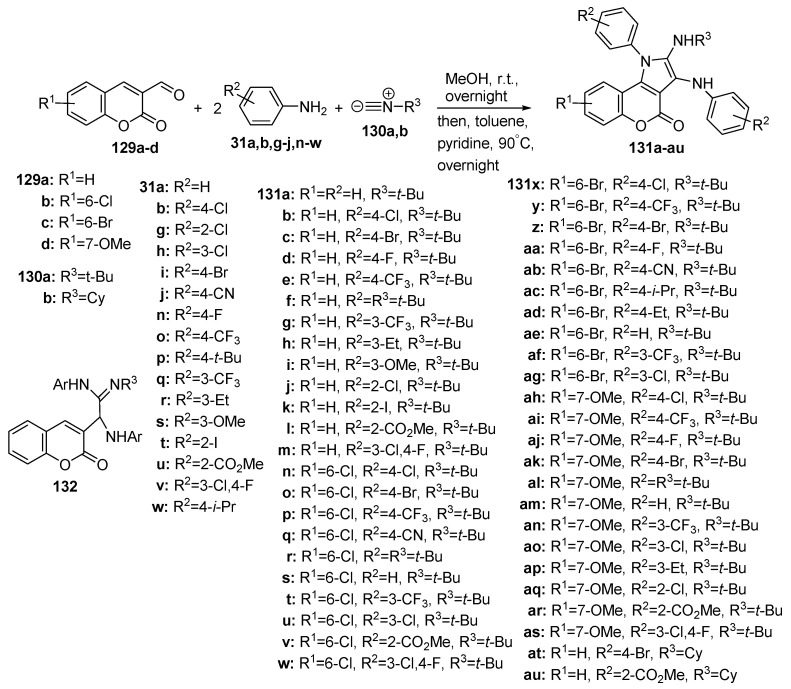
Synthesis of chromeno[4,3-*b*]pyrrol-4(H)-ones **131a**–**au** via one-pot multicomponent reaction.

Lai and Che synthesized substituted chromeno[4,3-*b*]pyrrol-4(*H*)-ones **131a**–**au** in 35–82% yield, via a one-pot MCR of 3-formylcoumarins **129a**–**d**, anilines (two equivalents) and isocyanides **130a**,**b** [105]. By the four-component reaction, an intermediate like **132** was formed, which through cyclization under Michael addition resulted in the final products (Scheme 38).

**Scheme 39:**
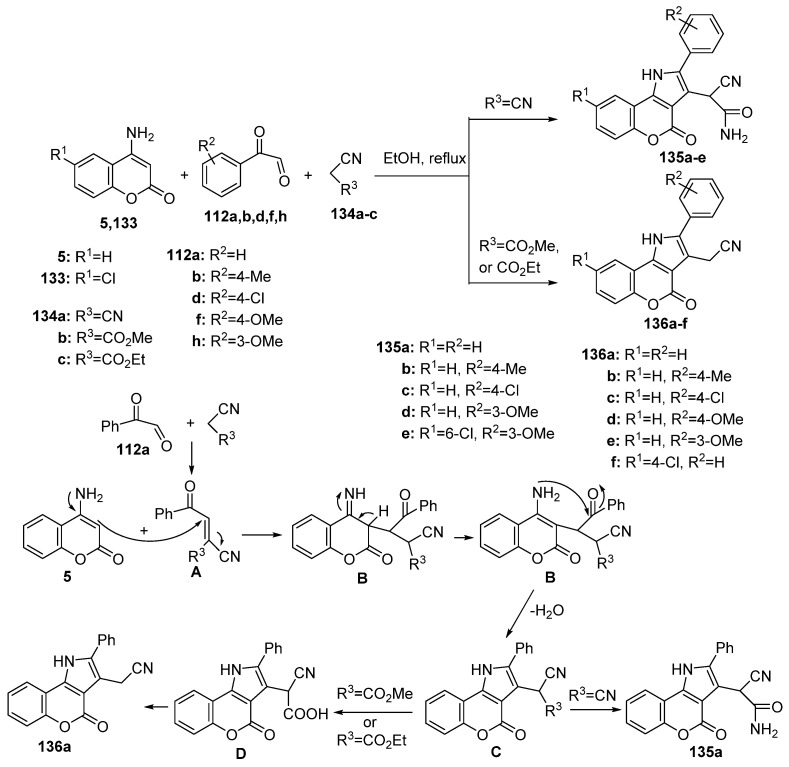
Synthesis of chromeno[4,3-*b*]pyrrol-3-yl derivatives **135a**–**e** and **136a**–**f**.

Recently, Karami et al. prepared chromeno[4,3-*b*]pyrrol-3-yl derivatives **135a**–**e** and **136a**–**f** in 90–98% yield through the one-pot MCR of aminocoumarins, aryl glyoxals and malono derivatives **134a**–**c** [106]. In the proposed mechanism, a Knoevenagel reaction of phenyl glyoxal (**112a**) with malono derivatives afforded the intermediate **A**. Michael addition of 4-aminocoumarin (**5**) to **A** furnished intermediate **B**, which tautomerized to **C**. The latter, in the case of malononitrile (**134a**), resulted in the final adduct **135a**. In the case of esters **134a**,**b**, hydrolysis furnished α-cyanoacid **D**, which upon elimination of CO_2_ afforded the nitrile derivative **136a** (Scheme 39). The above received pyrrolocoumarin derivatives were evaluated as α-glucosidase inhibitors. Most of the compounds presented potent inhibitory activity with IC_50_ = 48.65–733.83 μM. Compound **136d** was the most potent with an IC_50_ = 48.65 μM, 19-fold more potent than the standard inhibitor acarbose. Furthermore, they found that the presence of *para*-methoxy or *para*-chloro substituent on the phenyl ring improved the inhibitory activity.

#### 2.1.4. Synthesis through Metal-Catalyzed Reactions

**Scheme 40:**
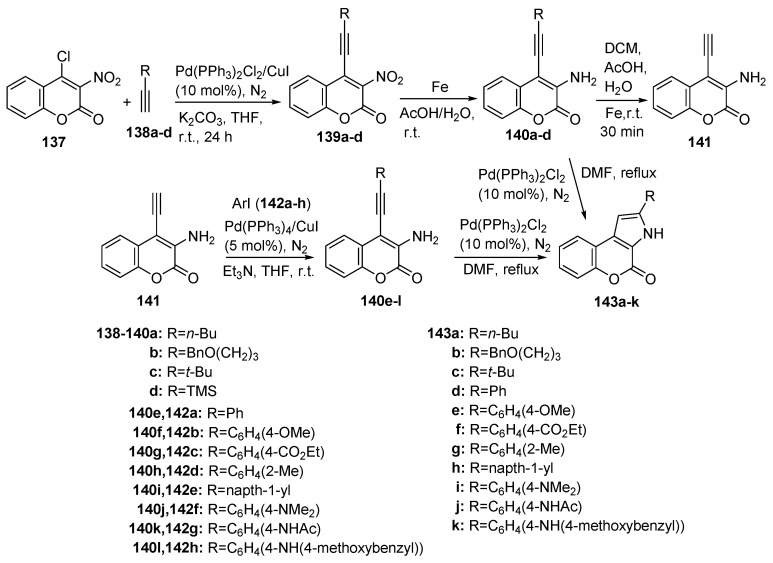
Pd-Catalyzed synthesis of chromeno[3,4-*b*]pyrrol-4(3*H*)-one derivatives **143a**–**k**.

The palladium-catalyzed synthesis of chromeno[3,4-*b*]pyrrol-4(3*H*)-one derivatives **143a**–**k** was presented by Chen and Hu and used further to synthesize lamellarin analogues [107]. The pyrrole ring was formed in 56–83% yield by the palladium-catalyzed intramolecular hydro-amination of 4-acetylenic 3-aminocoumarins **140**, synthesized during the reduction of 4-acetylenic 3-nitrocoumarins **139a**–**d** or by Sonogashira coupling of **141** with aryl iodides **142a**–**h** in 75–90% yield (Scheme 40). Desilylation of compound **140d** led to the 4-ethynyl-3-aminocoumarin (**141**). The 4-acetylenic coumarin derivatives **139a**–**d** were obtained by the Sonogashira coupling of 4-chloro-3-nitrocoumarin (**137**) with acetylene derivatives **138a**–**d**. 

**Scheme 41:**
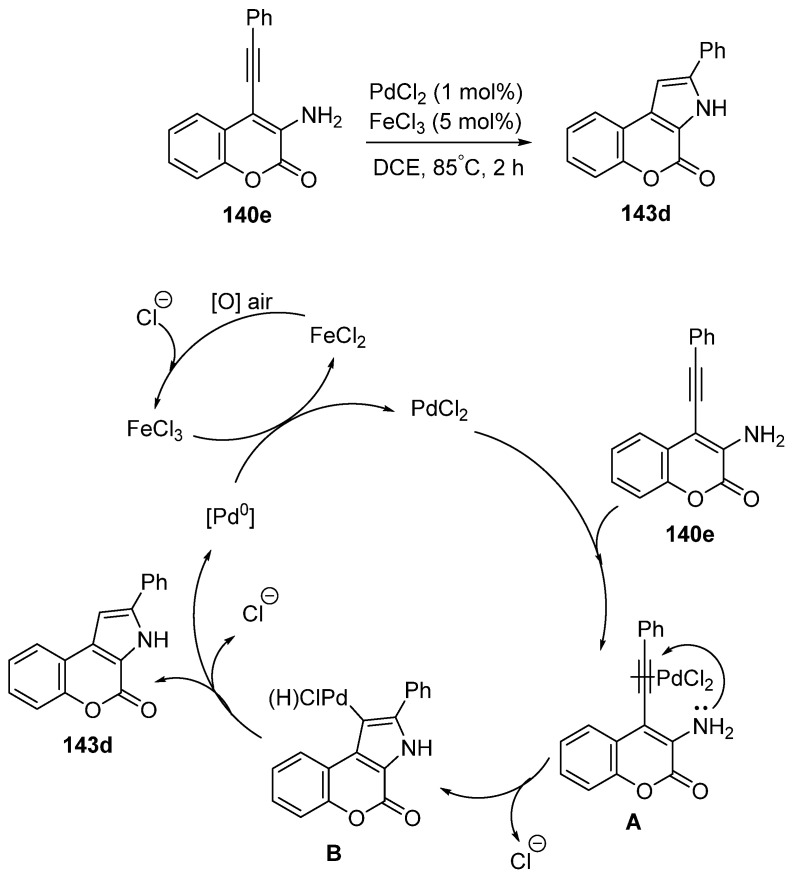
Iron/Palladium catalyzed synthesis of 2-phenylchromeno[3,4-*b*]pyrrol-4(3*H*)-one (**143d**).

Majumdar et al. prepared 2-phenylchromeno[3,4-*b*]pyrrol-4(3*H*)-one (**143d**) from 3-amino-4-(phenylethynyl)-2*H*-chromen-2-one (**140e**) under PdCl_2_ catalysis in the presence of FeCl_3_ along with other [5,6]-fused pyrrolocoumarin derivatives [108]. In the proposed mechanistic scheme, Pd(II) species coordinated with the triple bond of **140e**, activating it to form intermediate **A** (Scheme 41). Nucleophilic attack of the amine to the electron-deficient triple bond through a 5-e*ndo*-dig cyclization furnished the organopalladium (II) complex **B**. Reductive elimination afforded the final product **143d**. PdCl_2_ was reactivated by oxidation of Pd (0) with FeCl_3_. Reoxidation of the intermediate FeCl_2_ by air resulted in the formation of FeCl_3_, used in the catalytic cycle.

**Scheme 42:**
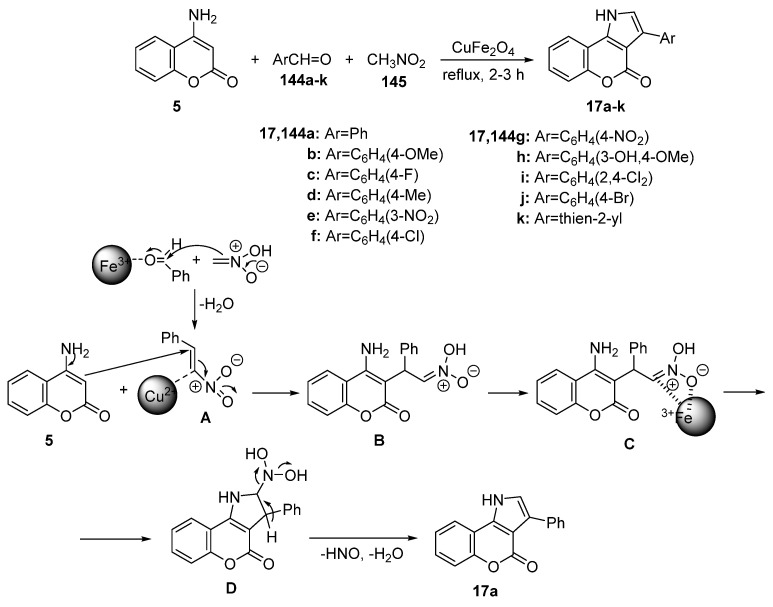
Preparation of chromeno[4,3-*b*]pyrrol-4(1*H*)-one derivatives **17a**–**k** through magnetic copper ferrite (CuFe_2_O_4_) nanoparticle catalysis.

Paul et al. utilized magnetic copper ferrite (CuFe_2_O_4_) nanoparticles for the three-component synthesis of chromeno[4,3-*b*]pyrrol-4(1*H*)-one derivatives **17a**–**k** in 81–95% yield via one-pot three-component domino reaction of 4-aminocoumarin (**5**), aromatic aldehydes **144a**–**k** and nitromethane (**145**) [109]. The catalyst was recycled six times, maintaining its catalytic activity. In the proposed mechanism, nitrostyrene **A** was formed through Knoevenagel reaction of benzaldehyde and nitromethane catalyzed by Fe^3+^ of CuFe_2_O_4_. The subsequent Michael addition of aminocoumarin **5** to **A** was catalyzed by Cu^2+^ of copper ferrite to give intermediate **B** (Scheme 42). By the interaction of Fe^3+^ with **B**, the electron-deficient complex **C** was generated, which upon electrophilic intramolecular cyclization resulted in the formation of dihydropyrrole-adduct **D**. Elimination of water and nitroxyl molecules afforded the final product **17a**.

**Scheme 43:**
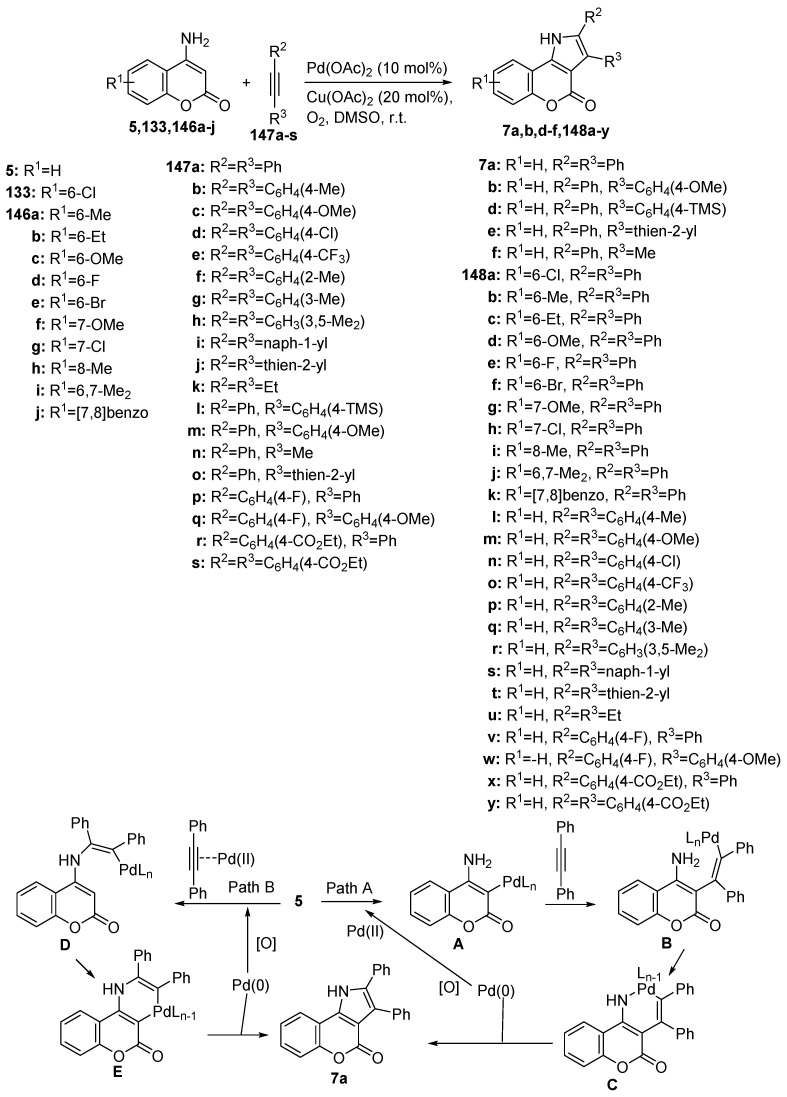
Synthesis of 2,3-disubstituted chromeno[4,3-*b*]pyrrol-4(1*H*)-one derivatives by the Pd-catalyzed oxidative reaction of 4-aminocoumarins with internal alkynes.

Peng et al. reported the synthesis of 2,3-disubstituted chromeno[4,3-*b*]pyrrol-4(1*H*)-one derivatives by the Pd-catalyzed, with Pd(OAc)_2_, oxidative reaction of 4-aminocoumarins with internal alkynes **147a**–**s** in the presence of Cu(OAc)_2_ under an atmosphere of oxygen [110]. The yields were good to excellent (60–99%). With different substituents of the internal alkynes, two regioisomers were obtained for compounds **7b**,**d**,**e** and **148v**,**w**. Two possible mechanistic schemes were proposed (Scheme 43). In path A, palladation of 4-aminocoumarin (**5**) afforded the palladium intermediate **A**. Subsequent *syn*-addition to diphenylacetylene generated the vinylpalladium species **B**, which by intramolecular palladation furnished palladium cyclohexene intermediate **C**. Reductive elimination in the latter resulted in the final product **7a**. In path B, alkyne was activated by Pd(II), acting as Lewis acid, and by the reaction with 4-aminocoumarin (**5**) afforded intermediate **D**. Electrophilic palladation of the latter under abstraction of proton furnished the intermediate **E**, which by reductive elimination resulted in the product **7a**. The generated Pd (0) was later oxidized to Pd (II), completing the catalytic cycle.

**Scheme 44:**
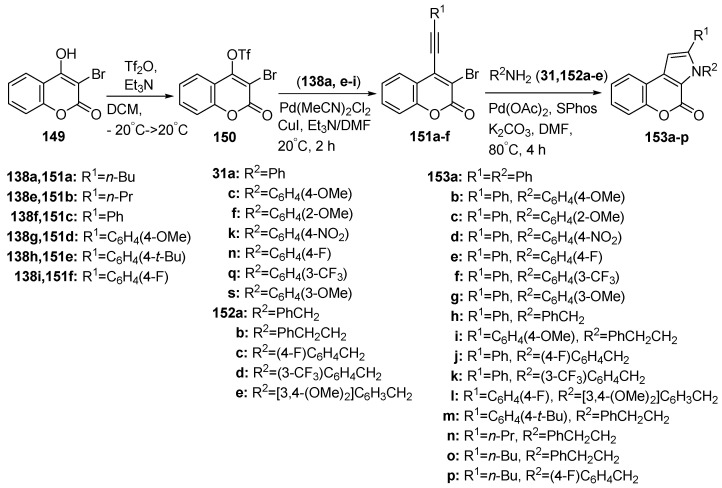
Synthesis of chromeno[3,4-*b*]pyrrol-4(3*H*)-ones by Pd-catalyzed domino C-N coupling/hydroamination reactions.

Ngo et al. synthesized chromeno[3,4-*b*]pyrrol-4(3*H*)-ones in 40–82% yield by the Pd-catalyzed domino C-N coupling/hydroamination reactions of 4-alkynyl-3-bromocoumarins **151a**–**f** with amines [111]. More nucleophilic anilines with electron-donating substituents resulted in better yields than anilines with electron-withdrawing substituents. Starting coumarins **151a**–**f** were isolated from the Sonogashira reaction of 3-bromo-4-(trifluoromethanesulfonyloxy) coumarin (**150**) with terminal alkynes (Scheme 44).

**Scheme 45:**
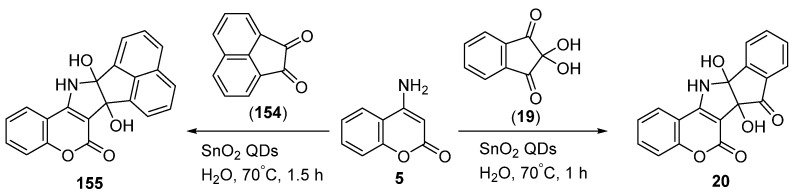
Synthesis of dihydroxy fused pyrrolo[3,2-*c*]coumarins containing indene or acenaphthene moieties by SnO_2_ quantum dot catalysis.

Pradhan et al. used nano-materials, the tin oxide quantum dots (SnO_2_ QDs), as a catalyst for the synthesis of dihydroxy fused pyrrolo[3,2-c]coumarins containing indene or acenaphthene moieties [112]. The reaction of 4-hydroxycoumarin (**5**) with ninhydrin (**19**) or acenaphthene quinone (**154**) resulted in the synthesis of compounds **20** and **155** in 90% and 83% yield, respectively (Scheme 45). The mechanism for these reactions follows a pattern similar to the one in Scheme 6.

**Scheme 46:**
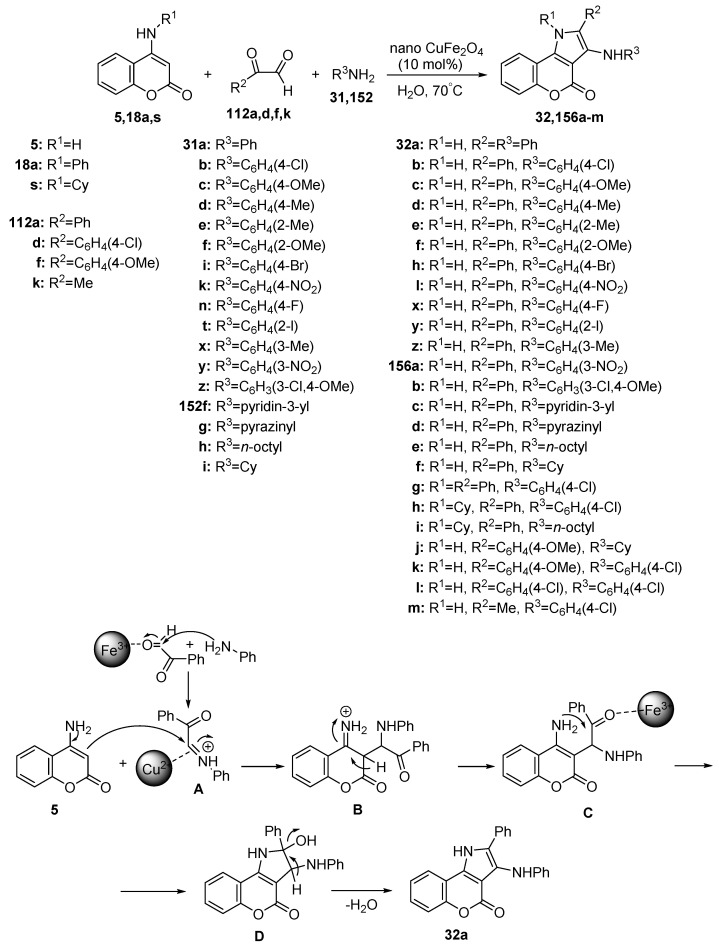
Synthesis of trisubstituted chromeno[4,3-*b*]pyrrol-4(1*H*)-ones by nano crystalline CuFe_2_O_4_ catalysis.

The research group of Dr. Das again used the earlier-referred nanocrystalline CuFe_2_O_4_ catalyst for the one-pot three-component reaction synthesis of chromeno[4,3-*b*]pyrrol-4(1*H*)-ones in 68–94% yield from 4-aminocoumarins, amines and aryl glyoxal monohydrates in water as solvent [113]. In the proposed mechanism, an imine intermediate **A** was formed from the reaction of amine with phenyl glyoxal monohydrate, activated by the Fe^3+^ center of the catalyst. Michael addition of 4-aminocoumarin (**5**) to **A**, catalyzed by Cu^2+^, afforded intermediate **B**. Tautomerization of the latter furnished intermediate **C**, which upon cyclization to **D** under Fe^3+^ catalysis, followed by elimination of water, resulted in the final product **32a** (Scheme 46). 

**Scheme 47:**
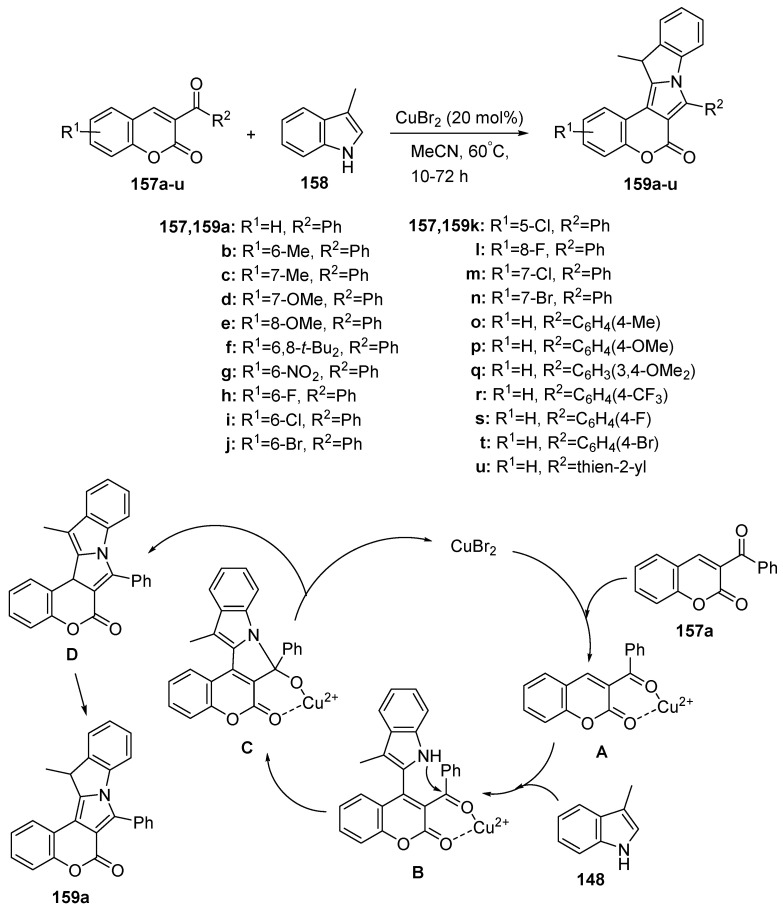
Synthesis of coumarin fused with 9*H*-pyrrolo[1,2-*a*]indoles.

Jin et al. presented the synthesis of coumarin derivatives fused with 9*H*-pyrrolo[1,2-*a*]indoles **159a**–**u** in 34–99% yield under a copper-catalyzed tandem reaction of 3-aroylcoumarins **157a**–**u** with 3-methylindole (**158**) [114]. In the proposed mechanism, 3-benzoylcoumarin was activated by CuBr_2_ to form an intermediate **A**. A Friedel–Crafts reaction of C-2-carbon of 3-methylindole (**158**) to **A** afforded intermediate **B**, which upon 1,2-addition of indole amine to benzoyl carbonyl furnished the cyclized adduct **C**. Dehydration of the latter resulted in intermediate **D**, releasing the catalyst. Isomerization of intermediate **D** afforded the final product **159a** (Scheme 47).

**Scheme 48:**
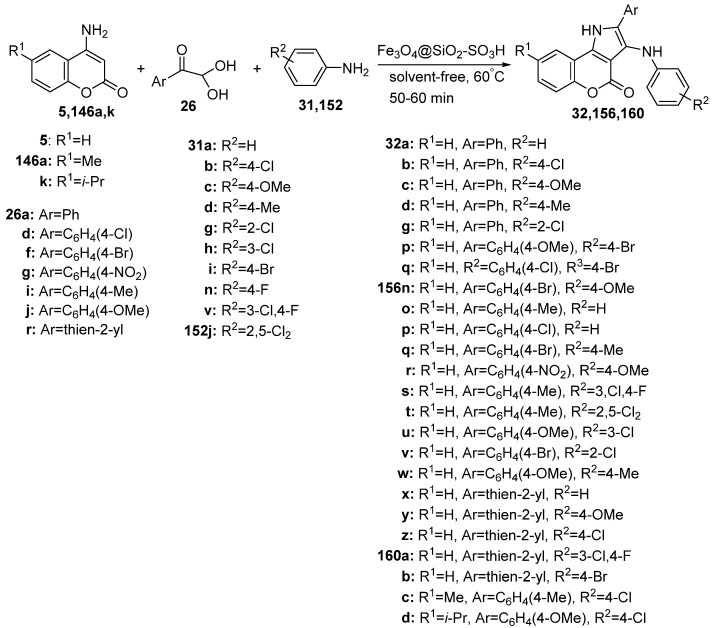
Synthesis of 3-arylaminochromeno[4,3-*b*]pyrrol-4(1*H*)-one derivatives using Fe_3_O_4_@SiO_2_-SO_3_H magnetic nanoparticles as catalyst.

**Scheme 49:**
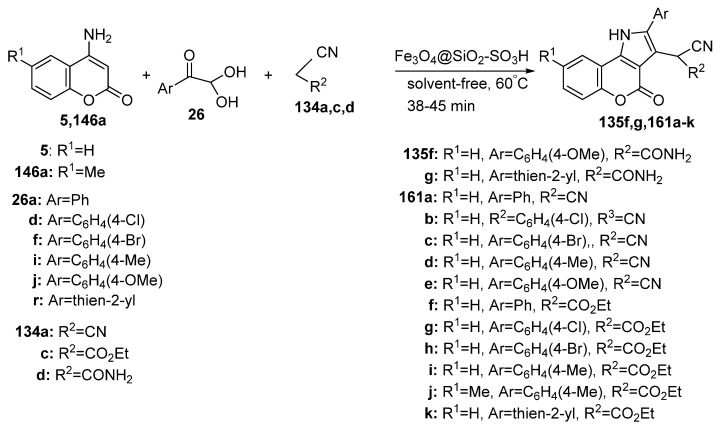
Synthesis of 3-cyanomethylenochromeno[4,3-*b*]pyrrol-4(1*H*)-one derivatives using Fe_3_O_4_@SiO_2_-SO_3_H magnetic nanoparticles as catalyst.

**Scheme 50:**
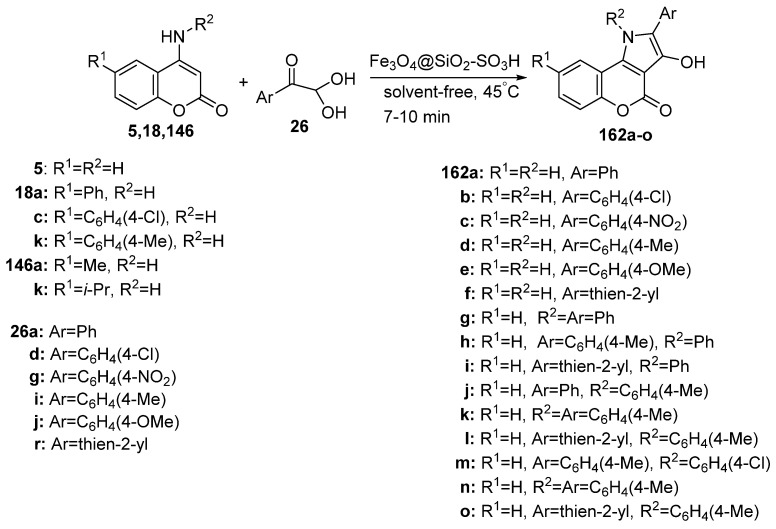
Synthesis of 3-hydroxychromeno[4,3-*b*]pyrrol-4(1*H*)-one derivatives using Fe_3_O_4_@SiO_2_-SO_3_H magnetic nanoparticles as catalyst.

**Scheme 51:**
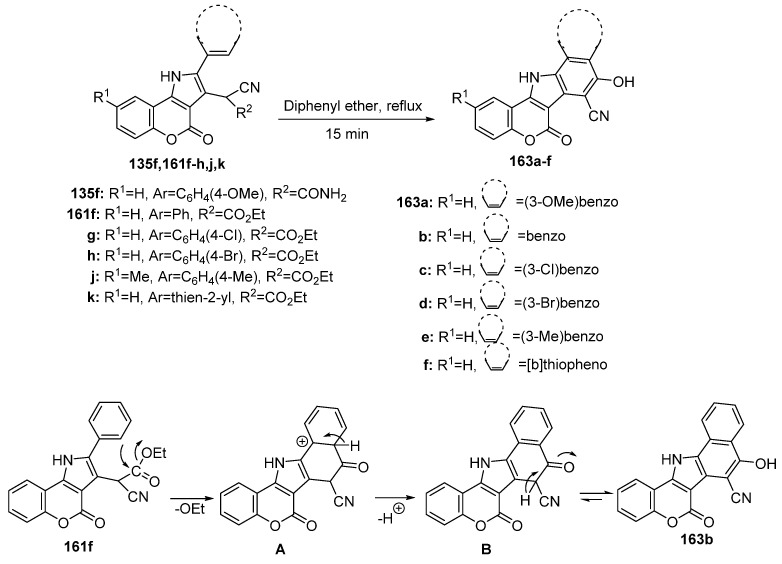
Synthesis of indolo[3,2-*c*]coumarin derivatives **163a**–**f**.

Mukherjee et al. used Fe_3_O_4_@SiO_2_-SO_3_H magnetic nanoparticles as catalyst under solvent-free conditions for the synthesis of chromeno[4,3-*b*]pyrrol-4(1*H*)-one derivatives substituted with arylamine, methylene nitrile, or hydroxy substituents in 59–80% yield (Scheme 48, Scheme 49 and Scheme 50). The mechanism for the synthesis of 3-arylamino- or 3-hydroxychromeno[4,3-*b*]pyrrol-4(1*H*)-one derivatives is similar to that presented in Scheme 31 with the initial formation of imino derivative or phenylglyoxal, respectively, from phenylglyoxal hydrate [115]. The mechanism for the formation of 3-cyanomethylenochromeno[4,3-*b*]pyrrol-4(1*H*)-one derivatives is analogous to that provided in Scheme 38. They also synthesized fused indolo[3,2-*c*]coumarin derivatives **163a**–**f** by refluxing of 3-cyanomethylenochromeno[4,3-*b*]pyrrolo-4(1*H*)-ones in diphenyl ether upon elimination of ethanol (from ethyl esters) or ammonia (from amide) (Scheme 51). 

**Scheme 52:**
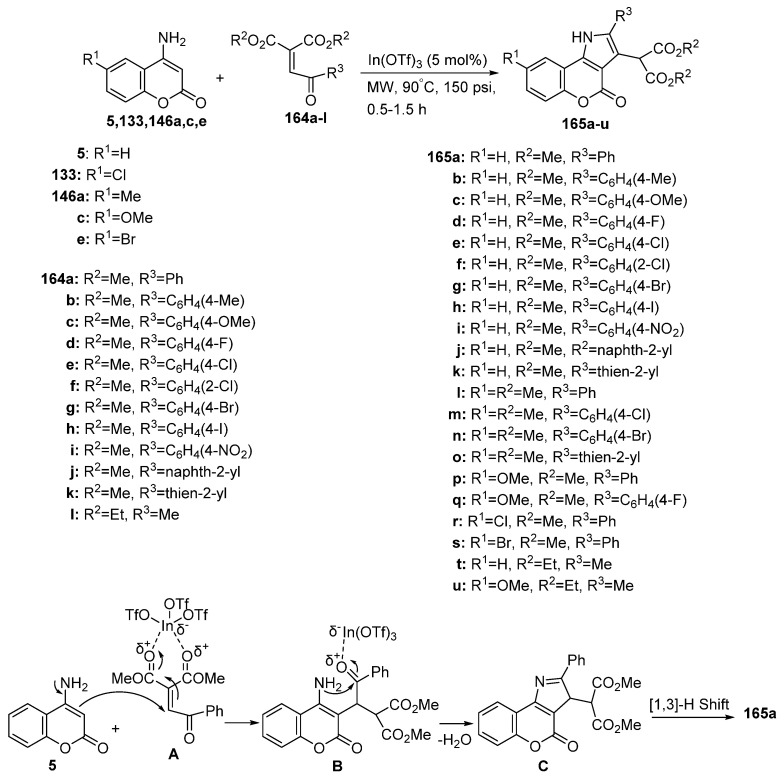
Synthesis of 3-disubstituted chromeno[4,3-*b*]pyrrol-4(1*H*)-one derivatives **165a**–**u** catalyzed by In(OTf)_3_.

Yadav et al. presented the synthesis of 2,3-disubstituted chromeno[4,3-*b*]pyrrol-4(1*H*)-one derivatives **165a**–**u** in 75–88% yield by the reaction of 4-aminocoumarins with aroylidene/acetylidene malonates **164a**–**l**, catalyzed by a Lewis acid, In(OTf)_3_, under microwave irradiation and solvent-free conditions [116]. In the possible mechanism, Michael addition of 4-aminocoumarin (**5**) to the reactive intermediate **A**, having lower LUMO energy due to coordination of **164a** with In(OTf)_3_, afforded adduct **B**. Cyclization of the latter under elimination of water to intermediate **C** and [1,3]-H shift resulted in the product **165a** (Scheme 52).

**Scheme 53:**
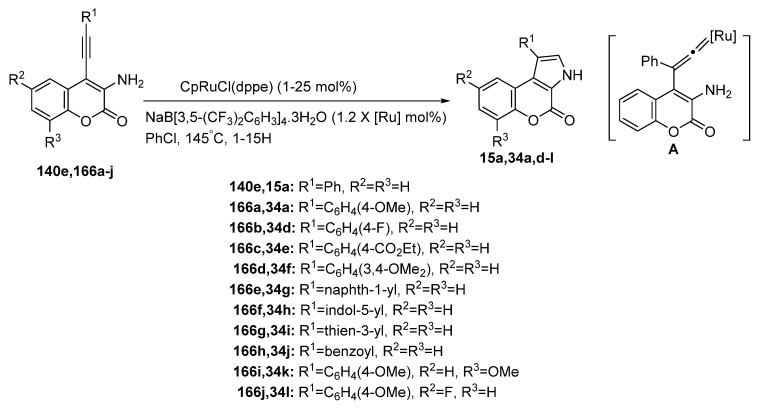
Synthesis of 1-arylchromeno[3,4-*b*]pyrrol-4(3*H*)-ones.

Watanabe et al. developed the preparation of 1-arylchromeno[3,4-*b*]pyrrol-4(3*H*)-ones in 81–98% yield by the ruthenium-catalyzed cycloisomerization of 4-alkynyl-3-amino-2*H*-chromen-2-ones **140**, **166** through 1,2-carbon migration [117]. For the mechanism of these reactions, a vinylidene rearrangement of internal alkyne by 1,2-carbon migration to form intermediate **A** (Scheme 53) and subsequent cyclization resulted in the final product **15a**. Additionally, they prepared Ningalin B and Lamellarin H by this method. 

**Scheme 54:**
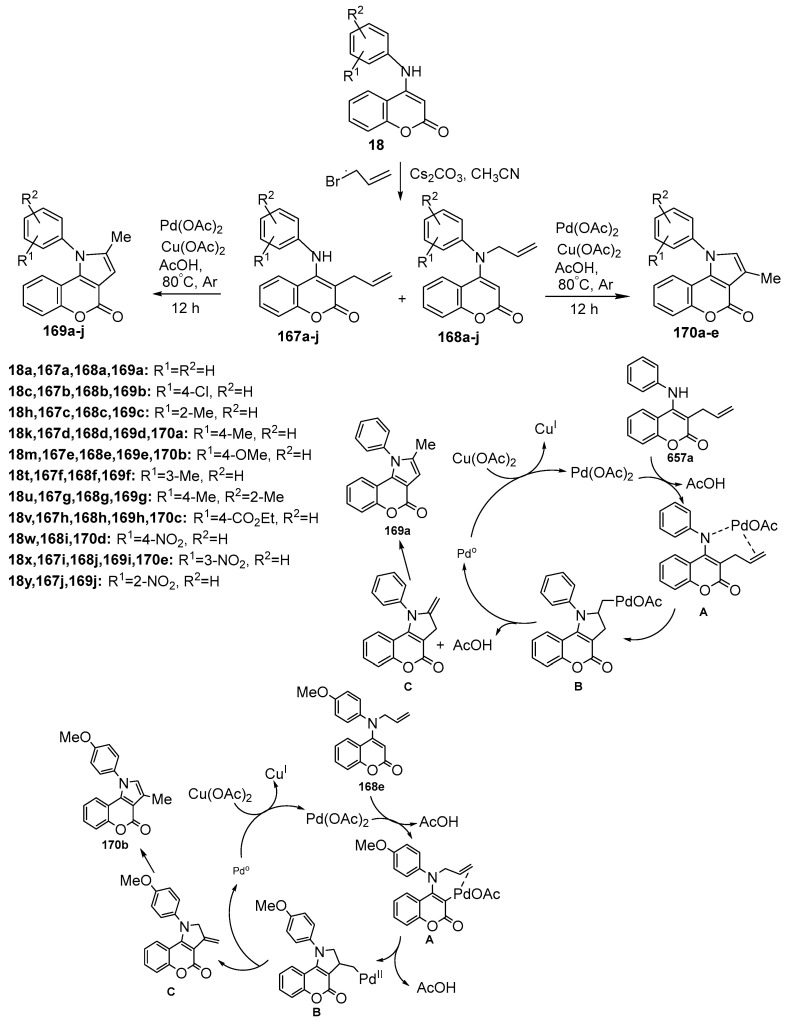
Synthesis of 1-aryl-2-methyl- or 3-methyl chromeno[4,3-*b*]pyrrol-4(1*H*)-ones catalyzed by palladium (II).

In 2022, our group reported the synthesis of 1-aryl-2-methyl- and 3-methyl chromeno[4,3-*b*]pyrrol-4(1*H*)-ones **169a**–**j** and **170a**–**e** in 91–95% yield by the Pd-catalyzed intramolecular Aza-Wacker-type cyclization of 3-allyl-4-arylaminocoumarins **169a**–**j** or C-H insertion/oxidative cyclization of N-allyl-N-aryl-4-aminocoumarins **168a**–**j**, respectively [46]. In the proposed mechanism, N-H activation of 1**67a** and coordination to double bond afforded intermediate **A**, which upon aminopalladation via 5-*exo-trig* cyclization furnished adduct **B**. Reductive elimination with β-hydride abstraction from the latter generated *exo*-methylene intermediate **C**, which through [1,3]-H shift resulted in the aromatic product **169a**. For the transformation of **168e** to **170b**, the palladated species **A** was formed, followed by insertion of double bond through 5-*exo-trig* cyclization to give intermediate **B**. Reductive elimination to **C** and tautomerization afforded the final product (Scheme 54). The above-prepared intermediate and final products presented interesting antioxidant activity while inhibiting soybean lipoxygenase (LOX). Compounds **159h**, **160b**, and **160a** presented the most interesting IC_50_ values (38, 41, and 45 μM, respectively) in LOX inhibition. The allyl-compounds **157e**, **157j**, and **158c** showed higher anti-LOX activities with IC_50_ values 10, 6, and 5.6 μM, respectively.

**Scheme 55:**
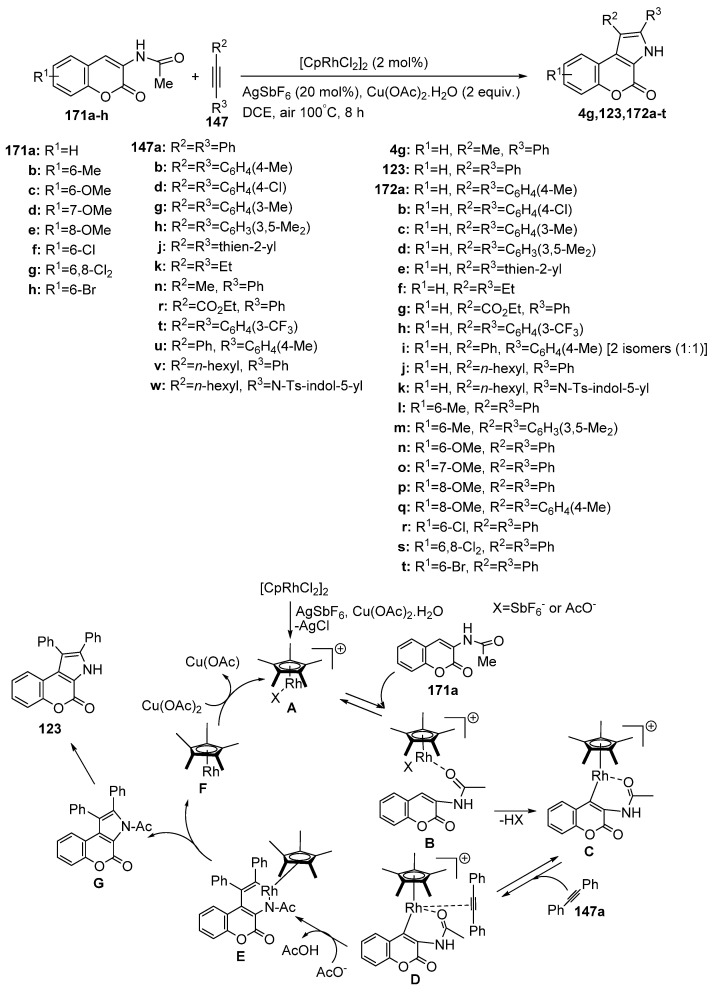
Synthesis of 1,2-disubstituted chromeno[3,4-*b*]pyrrol-4(3*H*)-one derivatives from 3-acetamidocoumarins and intermediate alkynes under Rh(III)-catalysis.

D. Das and A. R. Das synthesized 1,2-disubstituted chromeno[3,4-*b*]pyrrol-4(3*H*)-one derivatives from 3-acetamidocoumarins **171a**–**h** and internal alkynes **147** under Rh(III)-catalyzed C-H activation reactions using Ac-group as traceless directing group [118]. In the proposed mechanism, the active metalating species **A** formed from the precatalyst [CpRhCl_2_]_2_ and by interaction with Lewis basic carbonyl oxygen afforded intermediate **B**. C-H Bond cleavage produced species **C**, which coordinated with alkyne **147a** to form complex **D** and Rhoda-cycle **E** in the presence of AcO^−^ (Scheme 55). Reductive elimination of the latter furnished N-acyl adducts **G**, which upon hydrolysis resulted in the final product **123**. They performed fluorometric studies and found that compound **172p** is capable of differentiating the noxious Cr(VI) ions from Cr(III) ions.

**Scheme 56:**
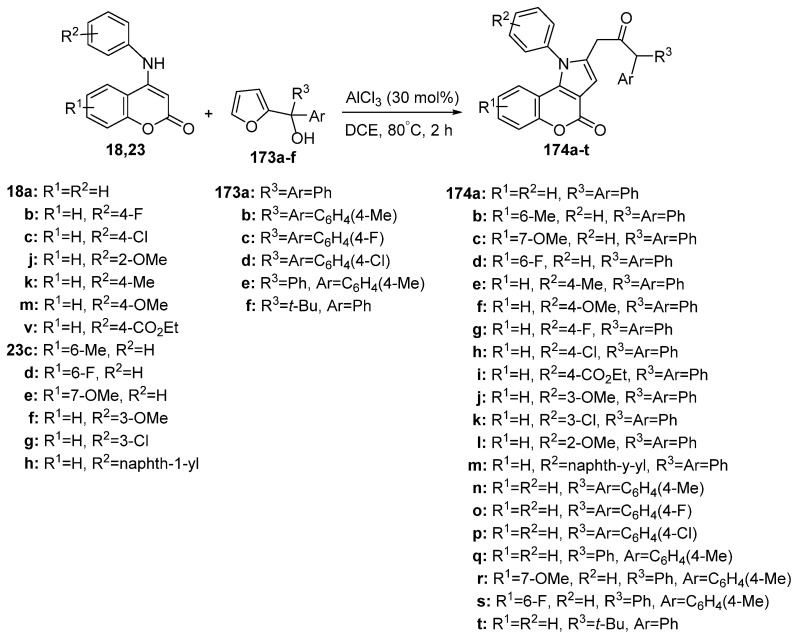
Synthesis of chromeno[4,3-*b*]pyrrol-4(1*H*)-ones **174a**–**t**.

Recently, Guan et al. presented the synthesis of chromeno[4,3-*b*]pyrrol-4(1*H*)-ones **174a**–**t** under AlCl_3_-catalyzed reactions of 4-anilinocoumarins with 2-furylcarbinols **173a**–**f** (Scheme 56). The reaction proceeded via chemoselective intermolecular α-carbon nucleophilic attack/ring-opening of furan ring/Michael addition/deprotonation aromatization processes [119].

### 2.2. Pyranone Ring Formation

**Scheme 57:**
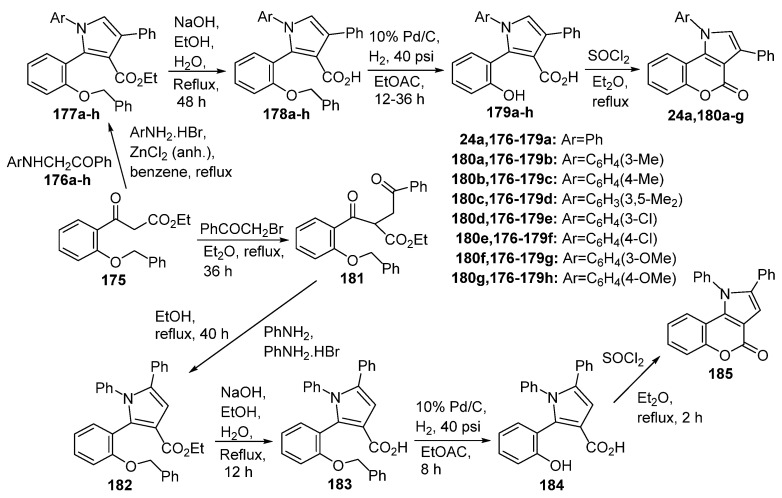
Synthesis of [1]benzopyranopyrrol-4-ones **24a**, **180a**–**g**, **185**.

In 1990 for the first time, Colotta et al. reported the synthesis of [1]benzopyranopyrrol-4-ones **24a**, **180a**–**g**, **185**, achieving the pyranone ring formation from pyrrole-3-carboxylic acids **179a**–**h**, **184** by refluxing in the presence of thionyl chloride (Scheme 57). The intermediate pyrrole derivatives **177a**–**h** were synthesized from ethyl 3-[2-(benzyloxy)-phenyl]-3-oxopropanoate (**165**) and phenacyl arylamines **176a**–**h**. Pyrrole-3-carbocylic acid **182** was obtained by the Paal–Knorr pyrrole synthesis from **181** and aniline. Compounds **24a**, **180a**–**g** were tested for their activity as BZR ligands [49]. These compounds demonstrated potent BZR binding activity, as they were examined for their ability to displace [^3^H]flunitrazepam from bovine brain membranes, showing IC_50_ values lower than that of diazepam (for **180c** and **180d**, IC_50_ = 8.6 and 10 μΜ, respectively; diazepam IC_50_ = 13 μΜ). The next year, the same group presented the synthesis of some different 1,3- and 1,2-disubstituted [1]benzopyrano-pyrrol-4-ones along with their BZR affinity, monitoring their ability to displace [^3^H]flunitrazepam [120].

**Scheme 58:**
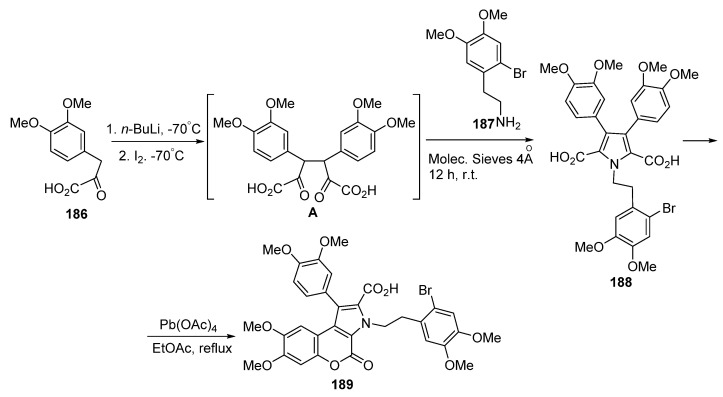
Synthesis of pyrrolo[2,3-*c*]coumarin derivative **189**.

The research group of Prof. Steglich synthesized the pyrrolo[2,3-*c*]coumarin derivative **189**, as intermediate for the synthesis of lamellarin G trimethyl ether in a biomimetic synthesis [121]. Oxidative coupling of two molecules of 3-(3,4-dimethoxyphenyl) pyruvic acid (**186**) furnished intermediate **A**, which reacted with 2-phenylethylamine **187** to give the pyrrole derivative **188** (Scheme 58). Treatment of **188** with Pb(OAc)_4_ afforded pyrrolo[2,3-*c*]coumarin derivative **189**. 

**Scheme 59:**
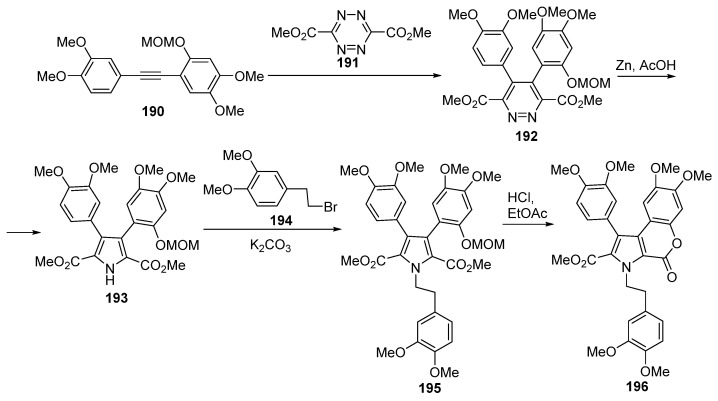
Synthesis of pyrrolo[2,3-*c*]coumarin derivative **195**.

Boger et al. used the heterocyclic azadiene Diels–Alder reaction for the synthesis of ningalin B [122] (Scheme 4). Diels–Alder reaction of ethynyl derivative **190** with tetrazine derivative **191** furnished diazine adduct **192**. Reductive ring contraction of the latter with zinc afforded the pyrrole **193**, which upon N-alkylation with aryl ethyl bromide **194** resulted in the compound **195** (Scheme 59). Lactonization of **195** furnished the pyrrolo[2,3-*c*]coumarin derivative **196**, intermediate for the synthesis of ningalin B. Compounds **195** and **196** were found to reverse the multidrug-resistant (MDR) phenotype, resensitizing a human colon cancer cell line (HCT116/VM46) to vinblastine and doxorubicin.

**Scheme 60:**
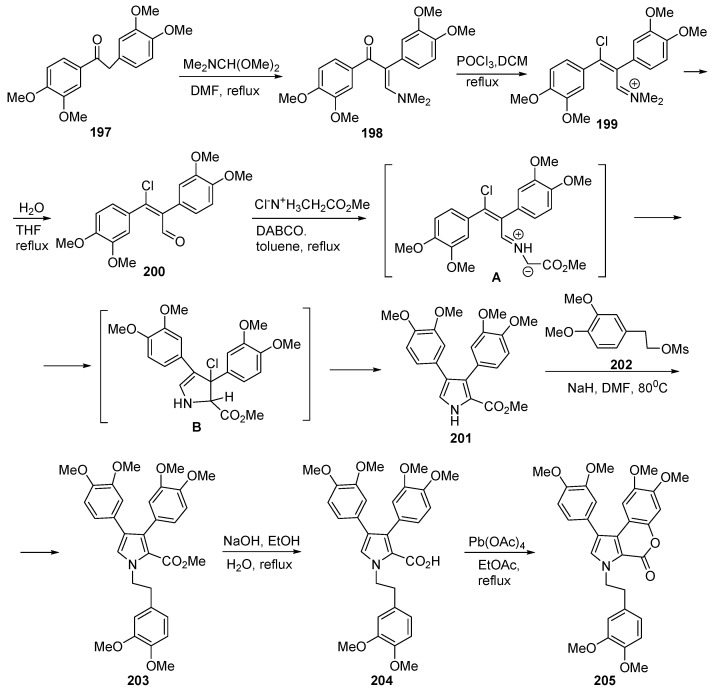
Synthesis of ningalin B hexamethyl ether (**205**).

Gupton et al. provided an alternative pathway for the synthesis of ningalin B hexamethyl ether (**205**) in 18% overall yield, acting as multidrug-resistant (MDR) phenotype reversal agent [123]. The trisubstituted pyrrole derivative **201** was prepared regioselectively, starting from desoxyveratroin (**197**) (Scheme 60). The latter, after reaction with *N,N*-dimethyl formamide dimethyl acetal, furnished enamine **198**, which upon chlorination with POCl_3_ to **199** and hydrolysis afforded β-chloroenal **200**. Reaction of **200** with glycine methyl ester hydrochloride resulted in the pyrrole **201** via the vinylogous iminium salt intermediate **A** and the dihydro pyrrole **B**. Alkylation of pyrrole **201** with mesylate ester **202** afforded *N*-aryl ethyl pyrrole **203**, which upon basic hydrolysis led to acid **204**. Oxidation of the latter with lead tetraacetate, as in Scheme 58, resulted in the pyrrolo[2,3-*c*]coumarin derivative, the ningalin B hexamethyl ether (**205**).

**Scheme 61:**
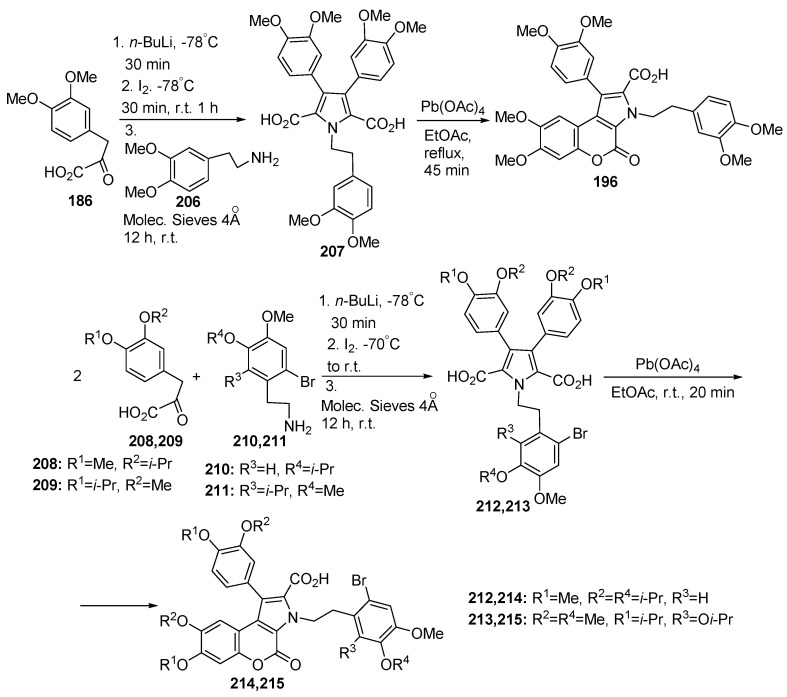
Synthesis of pyrrolo[2,3-*c*]coumarin derivatives **196**, **214**, **215**.

Peschko et al. developed the synthesis of marine alkaloids ningalin B, lamellarin G and lamellarin K utilizing as a key step in their earlier-applied method for the formation of 3,4-diarylpyrrole-2,5-dicarboxylic acids **207**, **212**, and **213** from arylpuruvic acids **186**, **208**, and **209** and 2-arylethylamines **206**, **210**, and **211**, respectively [124]. Oxidation of acids **207**, **212**, and **213** by led to tetraacetate-furnished pyrrolo[2,3-*c*]coumarin derivatives **196**, **214**, and **215** (Scheme 61), which are precursors for the synthesis of ningalin B, lamellarin G and lamellarin K, respectively. 

**Scheme 62:**
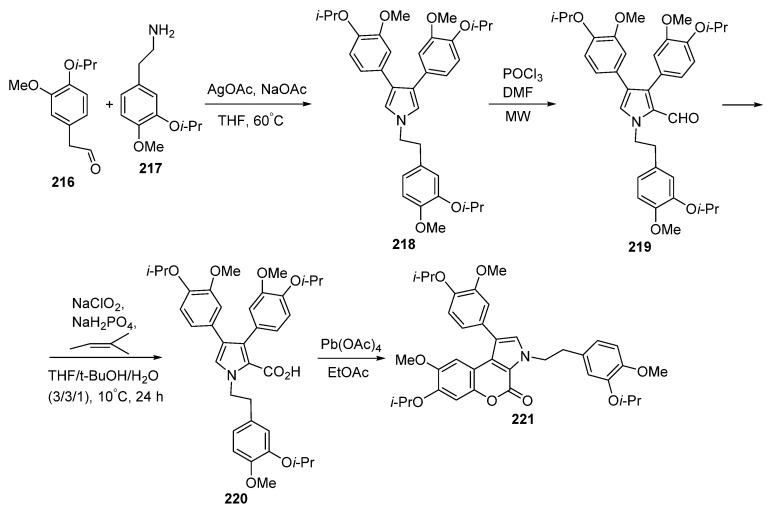
Synthesis of pyrrolo[2,3-*c*]coumarin derivative **221**.

Li et al. reported the total synthesis of lamellarins D and H and ningalin B from 2-(4-isopropoxy-3-methoxyphenyl) acetaldehyde (**216**) and 2-(3-isopropoxy-4-methoxyphenyl) ethylamine (**217**) [125]. The key steps for this procedure are three oxidative reactions, first involving AgOAc-mediative oxidative coupling of **216** with **217** to form pyrrole derivative **218**, followed by a microwave-accelerated Vilsmaier–Haack reaction to aldehyde **219**, which by Lindgren oxidation at 10 °C afforded the acid **220** (Scheme 62). A second oxidative cyclization of the latter with Pb(OAc)_4_ furnished pyrrolo[2,3-*c*]coumarin compound **221**, which was the intermediate for the synthesis of lamellarins D and H and ningalin B.

**Scheme 63:**
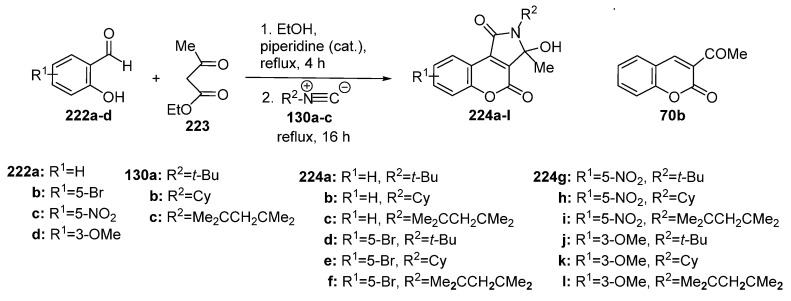
Synthesis of 5-hydroxy-N-substituted-2*H*-dihydrochromeno[3,4-*c*]pyrrole-2-ones **224a**–**l**.

Ghandi et al. reported the synthesis of 5-hydroxy-*N*-substituted-2*H*-dihydrochromeno[3,4-*c*]pyrrol-2-one derivatives **224a**–**l** in 52–65% yield by the three-component reactions of salicylaldehydes **222a**–**d** with ethyl acetoacetate (**223**) and isonitriles **130a**–**c** (Scheme 63). They found that the coumarin ring, like compound **70b**, was formed in situ during the reaction of salicylaldeyde (**222a**) with ethyl acetoacetate (**223**) [126].

**Scheme 64:**
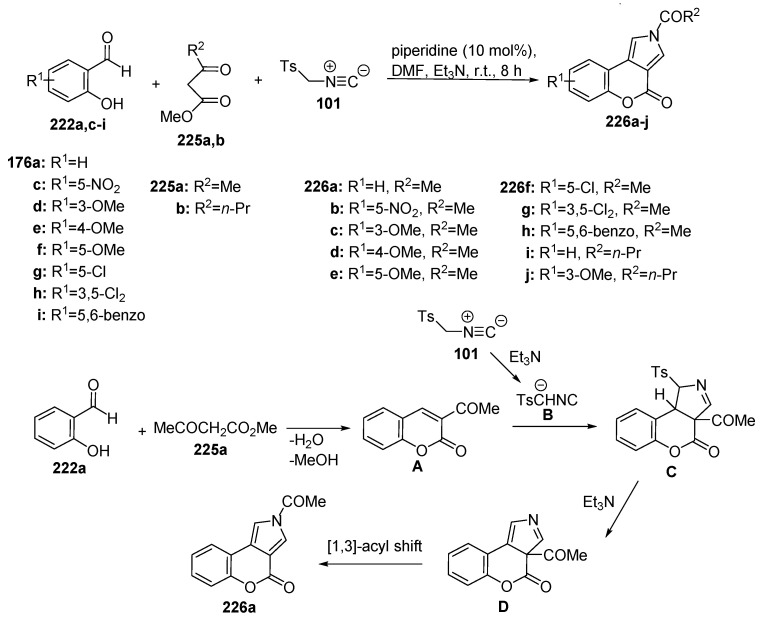
Synthesis of 2-acylchromeno[3,4-*c*]pyrrol-4(2*H*)-one derivatives **226a**–**j**.

Alizadeh et al. reported the preparation of 2-acylchromeno[3,4-*c*]pyrrol-4(2*H*)-one derivatives **226a**–**j** in 62–95% yield by the one-pot, three-component reaction of salicylaldehyde **222** with β-keto esters **225a**,**b** and tosylmethyl isocyanide (TosMIC) (**101**) [127]. In the plausible mechanism, Knoevenagel condensation of salicylaldehyde (**222a**) with methyl acetoacetate (**225a**) afforded 3-acetylcoumarin **A**. [3 + 2] Cycloaddition of TosMIC anion **B** to **A** furnished intermediate **C**. Desulfonylation of **C** with triethylamine produced intermediate **D**, which upon [1,3]-acyl shift resulted in the product **226a** (Scheme 64).

**Scheme 65:**
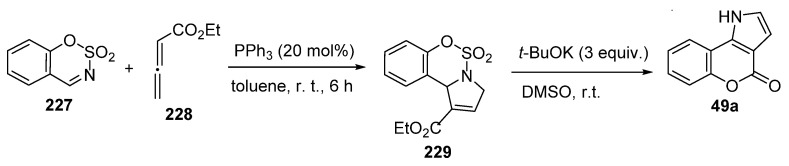
Synthesis of chromeno[4,3-*b*]pyrrol-4(1*H*)-one (**49a**) by treating sulfamate-fused dihydropyrrole (**229**).

Yu et al. isolated chromeno[4,3-*b*]pyrrol-4(1*H*)-one (**49a**) in 36% yield by treating sulfamate-fused dihydropyrrole (**229**) with *t*-BuOK [128]. Compound **229** was prepared by the reaction of sulfamate-derived cyclic imine **227** with ethyl allenoate **228** catalyzed by PPh_3_ (Scheme 65). 

**Scheme 66:**
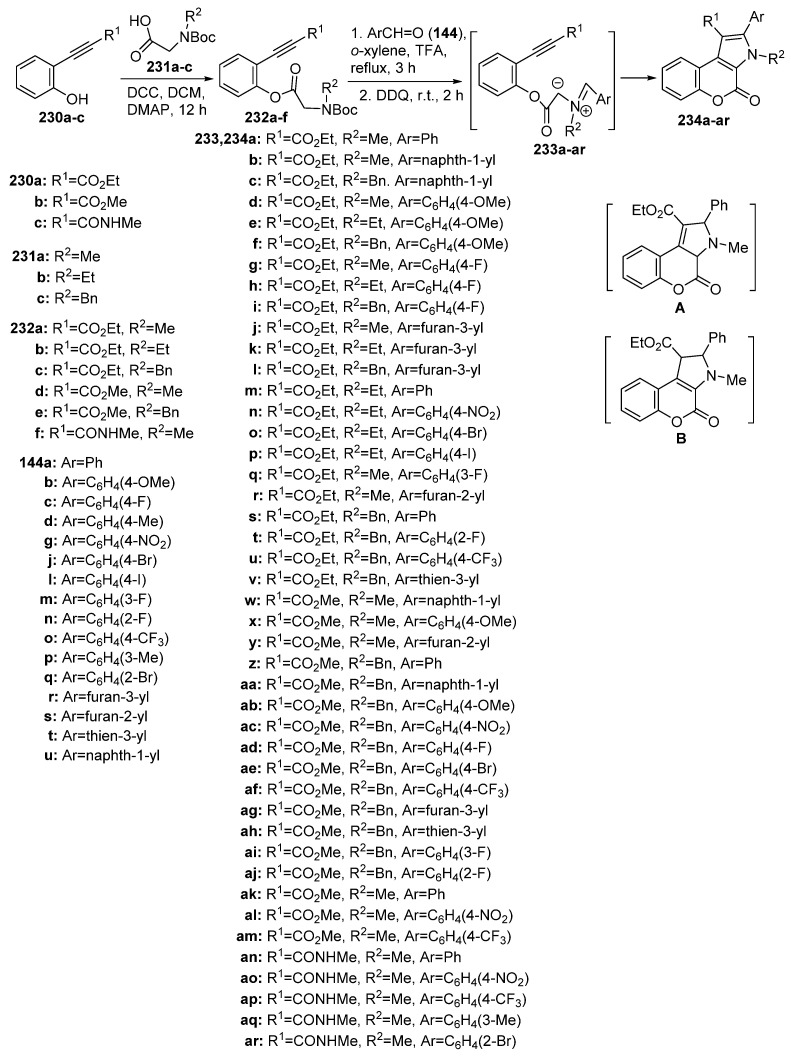
Synthesis of chromeno[3,4-*b*]pyrrol-4(3*H*)-one derivatives **234a**–**ar**.

Vidadala and Waldmann synthesized trisubstituted chromeno[3,4-*b*]pyrrol-4(3*H*)-one derivatives **234a**–**ar** in 48–88% yield by the one-pot intramolecular 1,3-cycloaddition reaction of azomethine ylides **233a**–**ar** [129]. The latter were prepared in situ from N-Boc-N-alkylated glycine esters **232a**–**f** and aromatic aldehydes **144** (Scheme 66). For the elucidation of the reaction sequence, they performed the deprotection of compound **232a** with TFA and the reaction of the produced adduct with benzaldehyde (**144a**) to isolate a mixture of dihydropyrrolocoumarines **A** and **B**. Oxidation of this mixture resulted in the product **234a**.

**Scheme 67:**
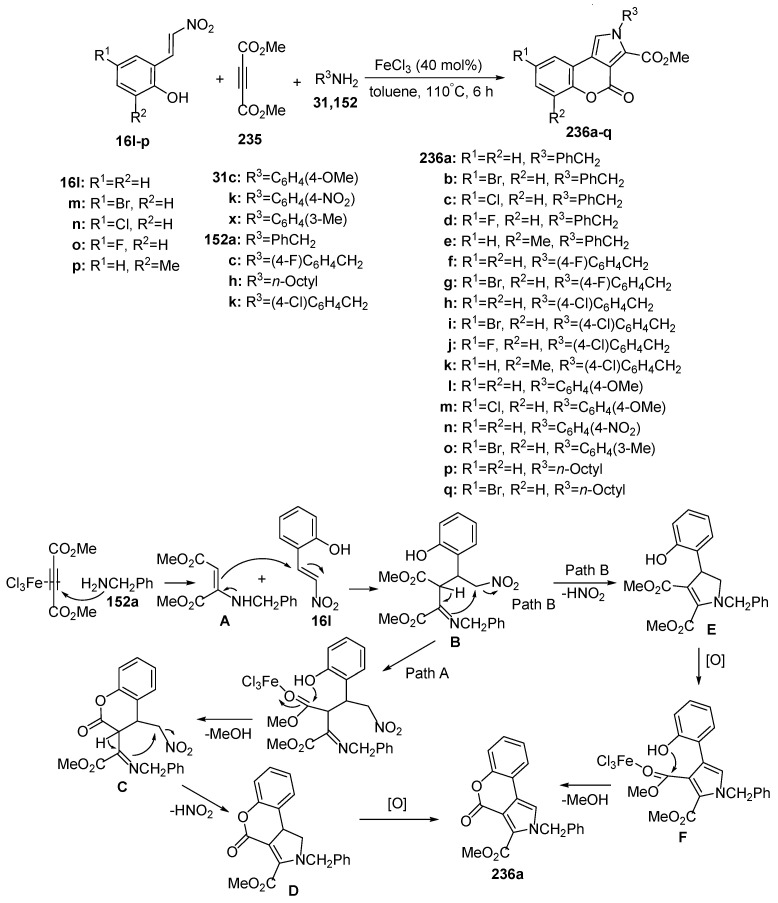
Synthesis of pyrrolo[3,4-*c*]coumarins **236a**–**q**.

Xue et al. developed the FeCl_3_-catalyzed synthesis of pyrrolo[3,4-*c*]coumarins **236a**–**q** in 50–92% yield by the three-component reaction of 2-(2-nitrovinyl)phenols **16l**–**p**, dimethyl acetylenedicarboxylate (**235**) and amines under formation of a pyranone ring [130]. In the proposed mechanism, nucleophilic addition of **152a** to **235** in the presence of FeCl_3_ generated intermediate **A**, which upon nucleophilic addition to **16l** afforded intermediate **B** (Scheme 67). FeCl_3_-mediated transesterification formed the pyranone ring with elimination of methanol in adduct **C**, during Path A. Subsequent nucleophilic attack with abstraction of nitro group furnished dihydropyrrole **D**, which upon air oxidation resulted in the product **236a**. In an alternative mechanism (Path B), intramolecular nucleophilic attack in **B** and elimination of nitro group afforded dihydropyrrole intermediate **E**. Air oxidation furnished pyrrole intermediate **F**, which by transesterification in the presence of FeCl_3_ resulted in the product **236a**. 

**Scheme 68:**
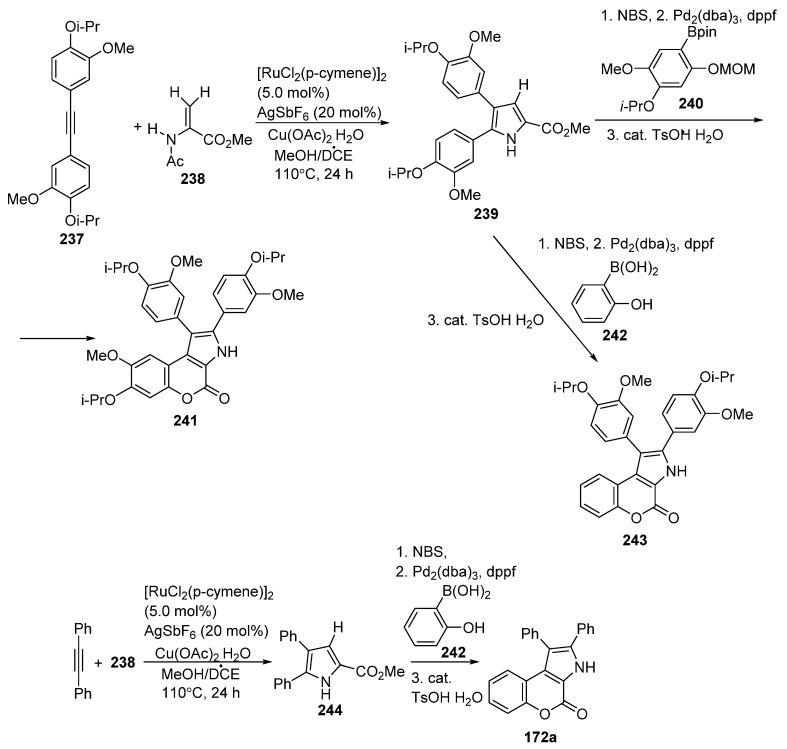
Synthesis of pyrrolo[2,3-*c*]coumarin derivatives **172a**, **241**, and **243**.

Ackermann and his colleagues presented the synthesis of lamellarin D and H precursors **241** and **243** [131]. The Ru-catalyzed C-H/N-H activation in an oxidation alkyne annulation process afforded the synthesis of pyrrole **239** from diaryl acetylene **237** and 2-aminoacrylate **238**. The Pd-catalyzed annulated formation of lactones **241** and **243** was achieved from **239** in a one-pot bromination/Suzuki cross-coupling procedure with NBS and boronates **240** and **242**, respectively (Scheme 68). 

**Scheme 69:**
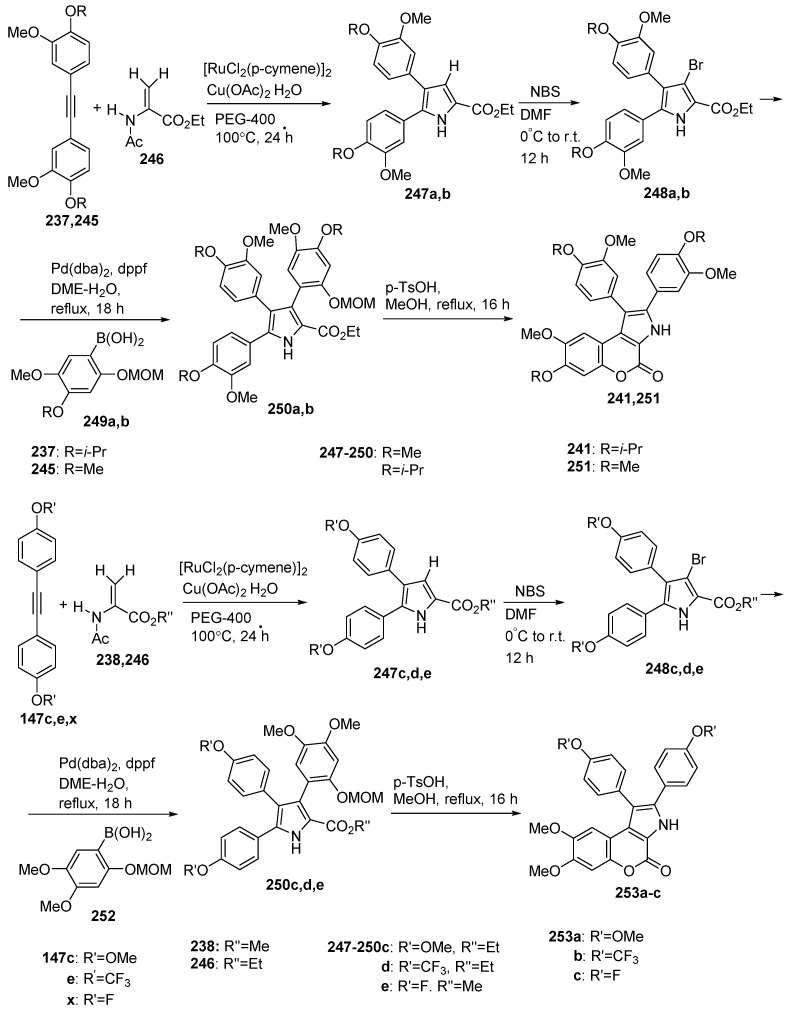
Synthesis of pyrrolo[2,3-*c*]coumarin derivatives **241**, **251**, and **253a**–**c**.

Lade et al. also used the Ru(II)-catalyzed strategy for the synthesis of pyrrole **250a**,**b** and pyrrolo[2,3-*c*]coumarin **241** and **251**, intermediates for the synthesis of lamellarin D, lamellarin D trimethyl ether, and lamellarin H [132]. The central pyrrole rings of compounds **247a**,**b** were prepared by the [3 + 2] cycloaddition reaction of enamide **246** with diaryl alkynes **237** and **245** under Ru-catalysis and in the presence of Cu(OAc)_2_.H_2_O as oxidant (Scheme 69). After bromination with NBS to bromo derivatives **248a**,**b**, followed by Suzuki–Miyaura cross-coupling with boronates **249a**,**b**, tetra-substituted pyrrole adducts **250a**,**b** were produced. Deprotection of the latter with TsOH in methanol and subsequent lactonization resulted in the preparation of pyrrolo[2,3-*c*]coumarin derivatives **241** and **251**. In an analogous process, pyrrolo[2,3-*c*]coumarins **253a**–**c** were prepared in 37–49% yield starting from diaryl alkynes **147c**,**e**,**x** and enamides **238** and **246**. 

**Scheme 70:**
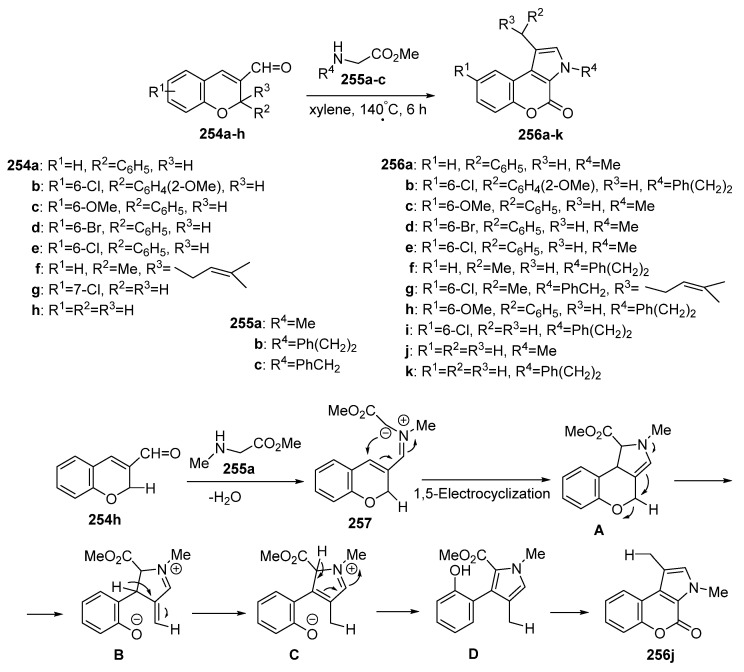
Synthesis of chromeno[3,4-*b*]pyrrol-4(3*H*)-ones **256a**–**k**.

Reddy et al. synthesized the chromeno[3,4-*b*]pyrrol-4(3*H*)-ones **256a**–**k** in 67–82% yield by the 1,5-electrocyclization of azomethine ylides derived from 3-formyl chromenes **254a**–**h** and N-alkyl glycine methyl esters **255a**–**c** [133]. In the proposed mechanism, the azomethine ylide **257**, formed from 3-formyl chromene (**254h**) and N-Me glycine methyl ester (**255a**) by 1,5-electrocyclization, led to the intermediate **A**, which by opening of the pyran ring afforded intermediate **B** (Scheme 70). Formation of an aromatic pyrrole ring in the intermediate **D**, followed by lactonization, resulted in the final product **256j**.

**Scheme 71:**
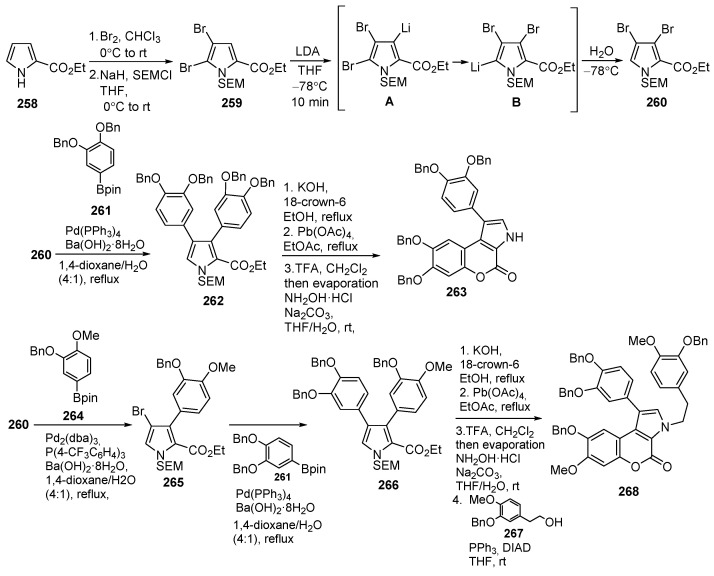
Synthesis of pyrrolo[2,3-*c*]coumarin derivatives **263** and **268**.

Morikawa et al. reported the synthesis of ningalin B and lamellarins S and Z using the dibromopyrrolo derivative **260** as starting material [134]. Compound **260** was prepared by bromination of ethyl 1*H*-pyrrole-3-carboxylate (**258**) followed by protection of nitrogen with 2-(trimethylsilyl)ethoxymethyl (SEM) to give **259** and subsequent treatment with LDA for the “halogen dance” via the unstable intermediates **A** and **B** (Scheme 71). Suzuki–Miyaura coupling of **260** with boronate **261** afforded the diarylated pyrrole ester **262**, which upon hydrolysis and Pb(OAc)_4_-mediated lactonization furnished pyrrolocoumarin derivative **263**, intermediate for the synthesis of ningalin B and lamellarin S. Regioselective Suzuki–Miyaura coupling of **260** with boronate **264** afforded, under monoarylation, pyrrole derivative **265**. Second Pd-catalyzed arylation of the latter with boronate **261** furnished diarylated pyrrole **266**. Similar treatment of **266** like the above-referred synthesis of compound **263** followed by Mitsunobu N-alkylation with aryl ethanol **267** resulted in the pyrrolocoumarin derivative **268**, which was the precursor for the synthesis of lamellarin Z. 

**Scheme 72:**
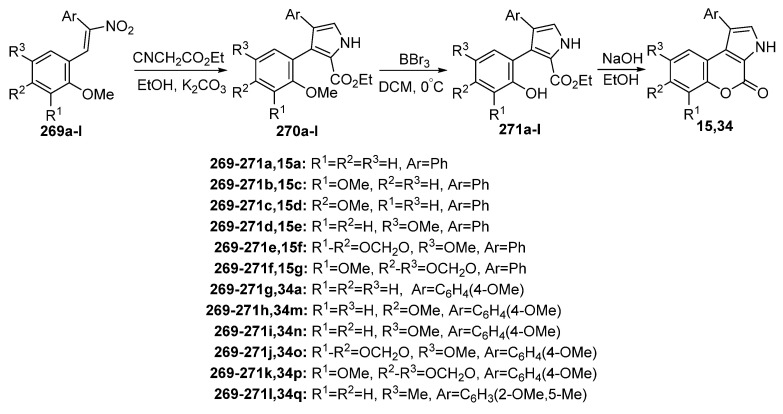
Synthesis of chromeno[3,4-*b*]pyrrol-4(3*H*)-one derivatives via Barton–Zard reaction and selective O-demethylation.

Silyanova et al. presented the synthesis of chromeno[3,4-*b*]pyrrol-4(3*H*)-one derivatives in 71–83% yield via selective *O*-demethylation of 3-(2-methoxyphenyl)pyrrole derivatives **270a**–**l** and treatment of the resulting phenolic derivatives **271a**–**l** with ethanolic NaOH at room temperature [135]. Compounds **270a**–**l** were prepared by the Barton–Zard reaction of nitrostilbenes **269a**–**l** with ethyl isocyanoacetate (Scheme 72).

**Scheme 73:**
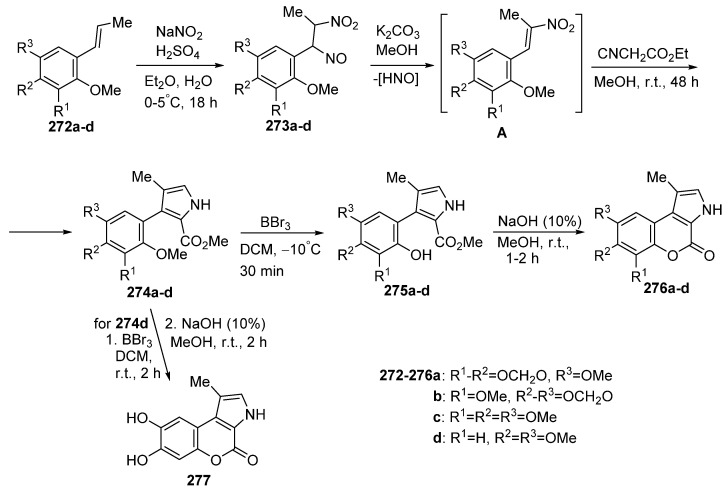
Synthesis of 1-methylchromeno[3,4-*b*]pyrrol-4(3*H*)-ones **276a**–**d** and **277**.

Rusanov et al. reported the synthesis of 1-methylchromeno[3,4-*b*]pyrrol-4(3*H*)-ones **276a**–**d** and **277** via the Barton–Zard reaction of nitroso compounds **273a**–**d** [136]. Compounds **273a**–**d** were synthesized from styrenes **272a**–**d** and, after the in situ formation of nitrostyrenes **A**, reacted with isocyano ethyl acetate to give trisubstituted pyrrole derivatives **274a**–**d** (Scheme 73). *O*-Demethylation of the latter with BBr_3_ followed by lactonization resulted in the synthesis of compounds **276a**–**d** in 9–31% yield from **272a**–**d**. When four equivalents of BBr_3_ were used in the case of **274d**, complete *O*-demethylation occurred to give dihydroxy derivative **277** in 72% yield.

**Scheme 74:**
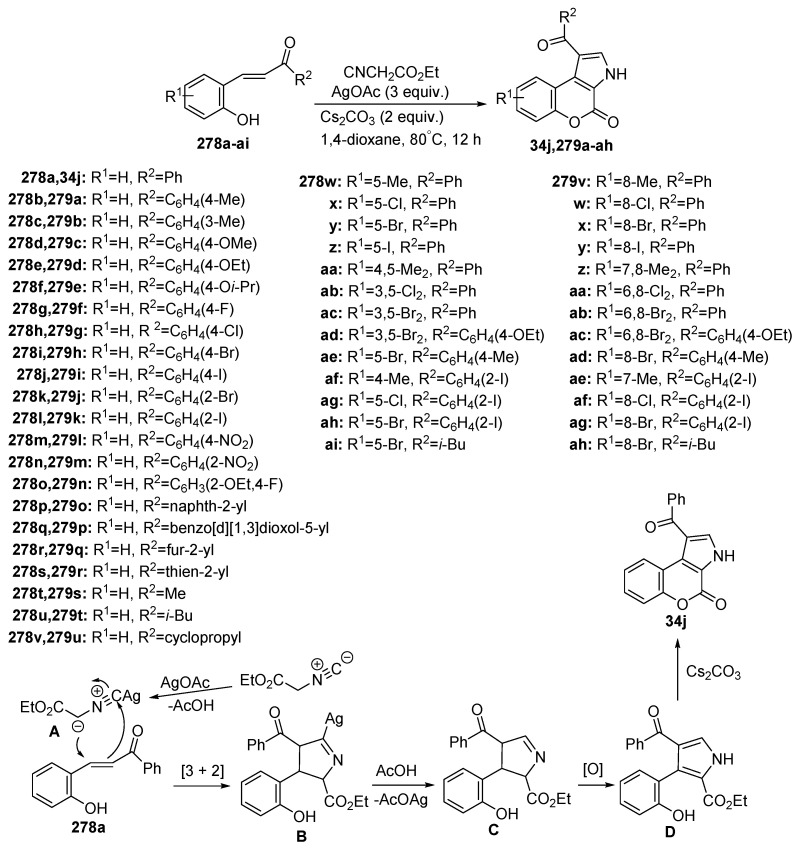
Synthesis of 1-ketylchromeno[3,4-*b*]pyrrol-4(3*H*)-one derivatives **34j**, **279a**–**ah** by tandem [3 + 2] cycloaddition/lactonization.

Recently, Das et al. reported the synthesis of 1-acylchromeno[3,4-*b*]pyrrol-4(3*H*)-one derivatives **34j**, **279a**–**ah** in 37–84% yield by the [3 + 2] cycloaddition reaction of ethyl isocyanoacetate with the double bond of 2-hydroxychalcones **278a**–**ai** followed by intramolecular lactonization, in a tandem process in the presence of AgOAc and Cs_2_CO_3_ [137]. In the proposed reaction mechanism, AgOAC with ethyl isocyanoacetate generated the 1,3-dipole **A**, which underwent [3 + 2] cycloaddition at the double bond of chalcone **278a**, affording intermediate **B**. Reaction of the latter with acetic acid furnished the intermediate **C**, which by tautomerization and oxidation afforded the pyrrole intermediate **D**. Intramolecular lactonization in the presence of Cs_2_CO_3_ resulted in the final product **34j** (Scheme 74).

**Scheme 75:**
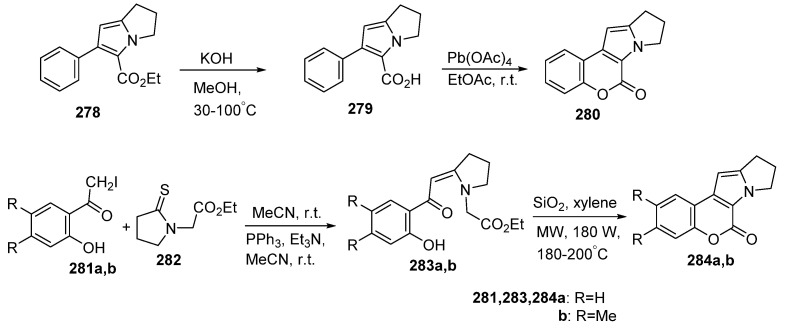
Synthesis of chromenone-fused pyrrolizines.

Very recently, Scalzullo et al. synthesized chromenone-fused pyrrolizines like **280** by oxidation of the corresponding pyrrole carboxylic acid **279** in low yield of 34% to prepare pyrrolizine-lamellarin analogues [138]. The phenolic enaminones **283a**,**b**, prepared by the reaction of iodide **281a**,**b** with thiolactam **282**, resulted in the preparation of 9,10-dihydrochromeno[4,3-*b*]pyrrolizin-6(8*H*)-ones **284a**,**b** in 86% yield after the formation of dihydropyrrolizine moiety, followed by lactonization (Scheme 75).

## 3. Conclusions

The present literature review showcases that the synthesis of 3,4-fused pyrrolocoumarins is achieved mainly by two routes. The formation of pyrrole moiety by one route is accomplished from a coumarin derivative, such as aminocoumarin, hydroxycoumarin, chlorocoumarin, or other coumarins. In the other route, a pyranone framework is formed from an existing pyrrole derivative. 1,3-Dipolar cycloaddition reactions, Michael reaction, aza-Claisen rearrangement reactions, MCR, as well as metal-catalyzed reactions are used in these syntheses. Name reactions, such as Fischer indole synthesis, Van Leusen type reaction, Ugi reaction, Aza-Wacker type cyclization, and Barton–Zard reaction, are useful for the synthesis of intermediates or final products.

Pyrrolocoumarins present cytotoxic, antifungal, antibacterial, α-glucosidase inhibition, antioxidant, lipoxygenase (LOX) inhibition, and fluorescent activities, and are used as BZR ligands. Especially, chromeno[3,4-*b*]pyrrol-4(3*H*)-one derivatives exhibited cytotoxic activity, and chromeno[4,3-*b*]pyrrol-4(1*H*)-one derivatives showed cytotoxic, fluorescence, antifungal, and lipoxygenase inhibitory activities or acted as BZR ligands, while 1-thiochromeno[3,4-*c*]pyrrole-3,4-dione derivatives revealed antibacterial and antifungal activities. 1,2-Dihydrochromeno[4,3-*b*]pyrrole-3,4-dione (**22**) was found to be 1.5-fold more toxic than 5-fluorouracil. 2-(2-(4-Methoxyphenyl)-4-oxo-1,4-dihydrochromeno[4,3-*b*]pyrrol-3-yl)acetonitrile (**136d**) was 19-fold more potent than acarbose in α-glucosidase inhibitory activity. 7-Methoxy-1,2-diphenylchromeno[3,4-*b*]pyrrol-4(3*H*)-one (**172p**) differentiated Cr(VI) ions from Cr(III) ions in fluorometric studies.

We want this review to benefit researchers in the field not only of pyrrolocoumarin derivatives but in coumarins in general.
